# Neural Mechanisms of Shooting Preparation Under High‐Risk and High‐Precision Tasks: A Multiscale EEG Study

**DOI:** 10.1002/brb3.71261

**Published:** 2026-03-09

**Authors:** Xinyu Shi, Xiuyan Hu, Xinzhou Chen, Aiyong Bao, Bowen Gong, Ting Shi, Yunfa Fu, Anmin Gong

**Affiliations:** ^1^ College of Information Engineering Engineering University of the People's Armed Police Force Xi'an China; ^2^ School of Information Engineering and Automation Kunming University of Science and Technology Kunming China; ^3^ School of Life Science and Technology Xi'an Jiaotong University Xi'an China

**Keywords:** EEG, functional connectivity, graph theory, shooting, stress, spectral analysis

## Abstract

**Background:**

During the shooting‐preparation phase, shooters frequently encounter multiple interfering factors, such as task load, social evaluation, and complex environments. These factors can induce intricate changes in neural activity, leading to variations in shooting performance. This study aims to investigate the neural mechanisms underlying brain activity during the shooting preparation phase in high‐risk tasks (e.g., Hostage‐Rescue Condition) and high‐precision tasks (e.g., Long‐Range Condition).

**Methods:**

Electroencephalographic (EEG) signals, the shooting performance metrics, and the self‐report measures were collected from 30 shooters who completed shooting tasks under three conditions: Hostage‐Rescue Condition, Long‐Range Condition, and Close‐Range Condition. EEG signals were subjected to sensor‐level spectral analysis, source‐level spectral analysis, functional connectivity analysis, and graph‐theoretic analysis.

**Results:**

Compared with Close‐Range Condition, shooters exhibited the following characteristics during Hostage‐Rescue Condition and Long‐Range Condition: (1) perceived pressure increased significantly; however, shooting score and aiming time improved significantly only in the Hostage‐Rescue Condition; (2) significant differences were observed across multiple frequency bands and brain regions. Sensor‐level spectral analysis revealed the greatest number of significant differences in beta‐band event‐related desynchronization/synchronization across conditions. Source‐space analysis indicated that the theta band exhibited the highest number of significant differences across conditions; (3) functional connectivity between the frontal lobe and other lobes weakened significantly, whereas intra‐lobar connectivity strengthened significantly. In addition, small‐worldness increased, but the clustering coefficient and global efficiency decreased significantly.

**Conclusion:**

Under Hostage‐Rescue Condition and Long‐Range Condition, shooters perceived greater pressure, yet the shooting score improved only under Hostage‐Rescue Condition. Intra‐lobar functional connectivity strengthened, whereas connectivity between the frontal lobe and other lobes was suppressed. Nodal clustering coefficients increased in vision‐related regions but decreased in semantically related regions. These changes indicate that, when confronting Hostage‐rescue and Long‐Range Conditions, the brain achieves adaptive regulation by redistributing neural resources to optimize information‐processing efficiency.

## Graphical abstract

This study investigated the neural mechanisms underlying the shooting preparation phase in 30 shooters across three task conditions, with concurrent collection of shooting performance metrics and self‐report measures. The findings revealed that during high‐pressure shooting tasks (Hostage‐Rescue and Long‐Range), shooters experienced significantly increased perceived pressure; however, shooting score improved only in the Hostage‐Rescue condition. EEG analysis showed reduced functional connectivity between the frontal lobe and other cortical regions, alongside enhanced intra‐lobar connectivity, indicating adaptive neural reconfiguration.

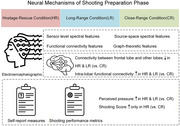

## 1 Introduction

Shooting skills, as a core competency in military, law enforcement, and competitive domains, directly affect mission success, personnel safety, and operational effectiveness through their precision, stability, and timeliness. In real‐world scenarios, individuals frequently encounter variable task loads, social evaluation, and complex environmental distractions. These factors can disrupt cognitive processing, decision‐making, and motor control, leading to performance fluctuations or operational errors. Therefore, an investigation of how such factors influence neural regulatory mechanisms during the shooting‐preparation phase is crucial for elucidating the neural basis of this stage, optimizing training protocols, and enhancing shooting performance.

Against this backdrop, electroencephalography (EEG) has emerged as a vital tool for studying cortical activity during the shooting‐preparation phase. Leveraging its millisecond temporal resolution and non‐invasive nature, EEG effectively captures neural oscillatory patterns closely linked to behavioral regulation.

Numerous studies have examined how different cognitive loads affect cortical activity and shooting performance. Kerick and Allender (2006) investigated the effects of target‐exposure duration, task load (single‐task vs. dual‐task), and decision load (friend‐or‐foe identification) on cortical activity, shooting accuracy, and subjective experience during a simulated shooting task. They found that shorter target‐exposure time significantly increased theta power at the Pz channel and decreased both shooting accuracy and decision‐making correctness. Dual‐task load and the requirement for friend‐or‐foe identification significantly reduced alpha power at the T7 channel. Thus, different types of cognitive load modulate shooting performance through specific oscillatory patterns in distinct frequency bands and brain regions (Kerick and Allender 2006).

Social‐evaluation‐induced performance pressure significantly alters cortical activity and shooting performance. Hatfield et al. (2013) and Woo and Kim (2017) compared cortical activity and motor performance between solo practice and real competitive scenarios. They found that, although competition did not significantly reduce hit rates, it markedly elevated participants' subjective stress levels, prolonged aiming time, and decreased whole‐brain alpha power while enhancing functional connectivity in the theta and alpha bands across regions including F3–F4, C3–C4, and T3–T4. These findings suggest that socially driven, non‐essential neural activity may impair psychomotor efficiency. Although prolonged aiming time temporarily preserves performance, this decline in efficiency may accumulate negative effects during extended training or high‐intensity tasks (Hatfield et al. 2013; Woo and Kim 2017).

Furthermore, complex environmental disturbances significantly alter neural activity patterns during the shooting‐preparation phase. Man et al. (2024) employed microstate analysis combined with source‐localization to investigate the neural mechanisms underlying pistol‐shooting preparation in 30 shooters under normal, dim, and noisy conditions. They found that, under dim and noisy conditions, microstate 3—associated with shooting—increased in strength, whereas microstate 4—reflecting attentional regulation—decreased; dim condition significantly reduced shooting performance, whereas noise condition had a smaller impact on performance (Man et al. 2024). Gong et al. (2025) extracted multiple complexity metrics based on microstate analysis and source localization to examine brain complexity during the shooting‐preparation phase and the neural response mechanisms under audiovisual constraints. They revealed that, during shooting, visual information processing relied primarily on secondary visual cortices and their connectivity rather than on the primary visual cortex. Noise had a minimal effect on shooting performance, whereas dim light had multifaceted adverse effects, including difficulties in sensorimotor integration, excessive reliance on memory retrieval, reduced motor stability, heightened negative emotions, and altered shooting strategies (Gong et al. 2025).

To further elucidate the intrinsic relationship between neural activity and shooting performance, some studies have examined the association between different levels of shooting performance and EEG characteristics. Cheng et al. (2023) categorized shooters into superior and inferior groups based on median shooting scores and compared their EEG activity during the shooting‐preparation phase. They found that the superior group exhibited stronger sensorimotor rhythms during preparation, which were positively correlated with reduced aiming jitter. During bullseye hits, they demonstrated reduced high‐frequency alpha‐band functional connectivity between the frontal and left temporal regions (Fz–T3), indicating more effective suppression of task‐irrelevant neural interference. These findings support the neural‐efficiency hypothesis, which posits that elite performance relies on selective activation of task‐relevant networks and effective inhibition of irrelevant regions (Cheng et al. 2023).

In summary, EEG research provides a crucial window into understanding the neural mechanisms underlying the shooting‐preparation phase as modulated by various factors; however, current knowledge remains incomplete. On the one hand, existing studies have focused primarily on experimental paradigms involving cognitive load, social evaluation, and complex environmental interference, whereas the neural mechanisms underlying high‐risk scenarios (e.g., Hostage‐Rescue Condition) and high‐precision task demands (e.g., Long‐Range Condition) remain underexplored. On the other hand, in terms of analytical methods, most studies are confined to the sensor level, with scarce research on functional connectivity and graph‐theoretic features after source localization.

Classical spectral analysis is susceptible to volume conduction effects (Nash et al. 2023). Source localization reconstructs cortical activity sources by solving inverse problems, thereby revealing more precise patterns of cortical activation. Brain network analysis offers deeper insights into functional brain states by quantifying functional connectivity between brain regions (Briels et al. 2020). Graph‐theoretic metrics, derived from functional connectivity networks, capture the topological properties of these networks and have been extensively applied in neuroscience research.

To address the aforementioned issues, this study proposes the hypothesis that high‐risk scenarios (e.g., Hostage‐Rescue Condition) and high‐precision task demands (e.g., Long‐Range Condition) significantly alter neural activity patterns during the shooting‐preparation phase. Enhanced activation in specific frequency bands and brain regions, along with alterations in functional connectivity within brain networks, subsequently impact shooting performance. To test this hypothesis, we established three shooting‐task conditions: Close‐Range Condition, Long‐Range Condition and Hostage‐Rescue Condition. Spectral features at the sensor level captured temporal changes in neural activity, while source localization explored spatial activation patterns across brain regions. Functional connectivity matrices were further computed and subjected to graph theory analysis to reveal functional interaction mechanisms between different brain areas. This multiscale approach aims to clarify how high‐risk scenarios and high‐precision tasks influence neural regulatory mechanisms and behavioral performance during the shooting‐preparation phase.

## 2 Methods

### 2.1 Experimental Design

The experiment recruited 35 male participants (aged 20–24, mean age = 22.1, SD = 0.9). All were enrolled at the Engineering University of the People's Armed Police Force, had completed the institution's shooting course, and had passed skill assessments. They were designated as “proficient shooters” by university experts on the basis of training duration and shooting performance. Inclusion criteria were good physical health; normal vision and hearing; no history of psychiatric or neurological disorders; right‐handedness with right‐hand shooting; and no consumption of stimulant beverages or neuroactive medications within 24 h before the experiment. After receiving a full explanation of the study objectives and procedures, all subjects provided written informed consent and participated voluntarily. In addition, all subjects completed the SCL‐90 psychological screening to exclude individuals with current significant symptoms of anxiety, depression, or post‐traumatic stress disorder (PTSD).

**FIGURE 1:**
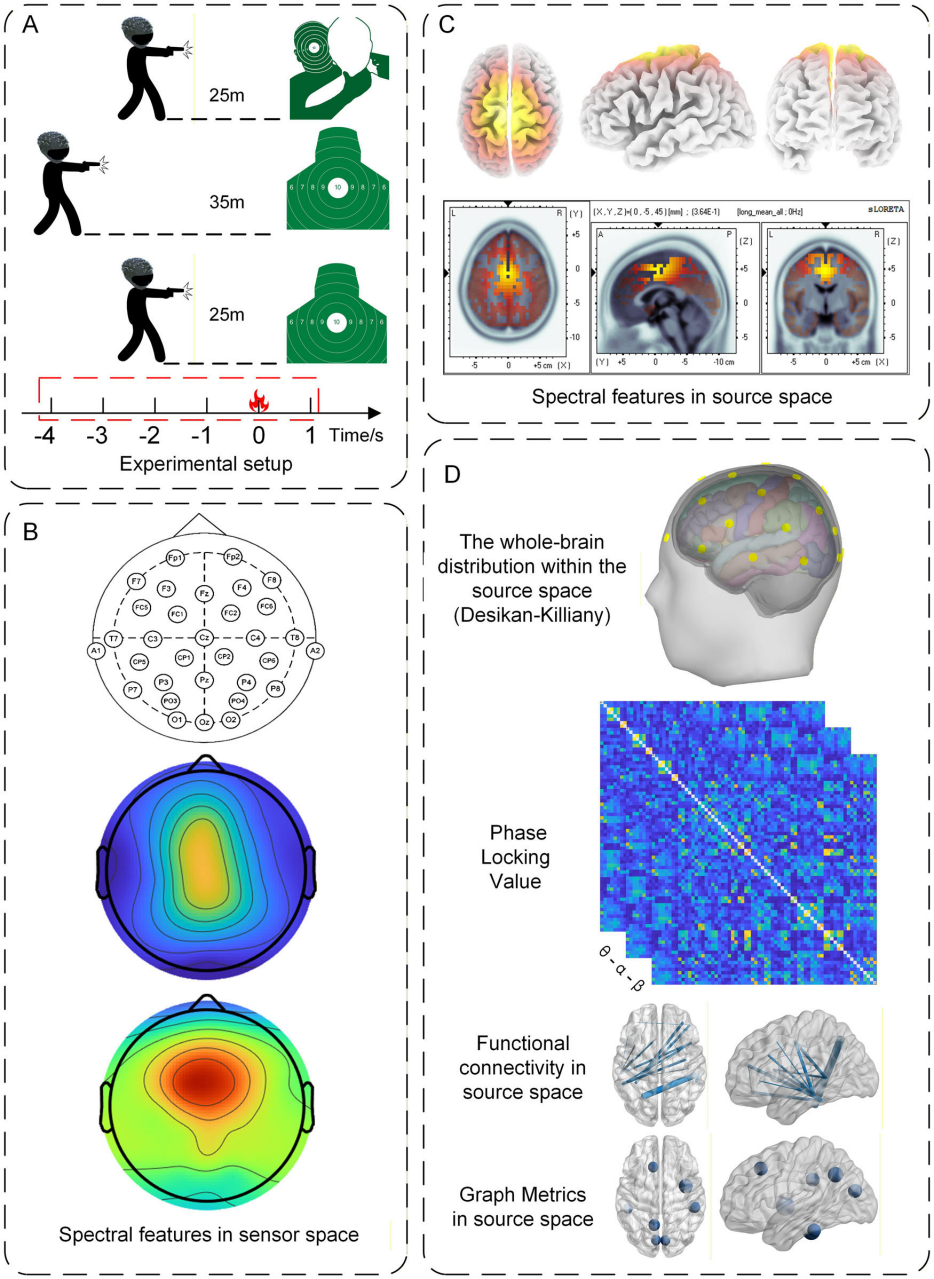
Experimental workflow. (A) Experimental setup; (B) sensor‐level spectral features; (C) source‐space spectral features; (D) functional‐connectivity and graph‐theoretic features.

Using a QSZ‐92 semi‐automatic pistol, each participant completed two blocks of self‐paced shooting under three conditions: Hostage‐Rescue Condition, Long‐Range Condition, and Close‐Range Condition, yielding 6 blocks of 30 shots each (180 shots in total). A 10‐min rest separated successive blocks, and the order of conditions was randomized. As illustrated in Figure 1A, Close‐Range Condition and Hostage‐Rescue Condition were conducted at 25 m, whereas Long‐Range Condition was performed at 35 m. Standard chest‐ring targets were employed for Close‐Range Condition and Long‐Range Condition; the Hostage‐Rescue Condition utilized a hostage‐scenario target—identical in ring size and scoring zones to the standard target—but depicting a hostage‐taker partially obscured behind a hostage. Scores were displayed immediately after each shot. In the Hostage‐Rescue Condition, a miss was recorded as “hostage death,” and if the mean shooting score fell below 8 rings, an additional block was required. Participants could withdraw at any time if they felt unwell. The protocol was approved by the Scientific Ethics Committee of the Engineering University of the People's Armed Police Force.

EEG was recorded with a 32‐channel wireless NSW332 amplifier (Borui Kang Technology, China) at a sampling rate of 1 kHz. Electrodes were positioned according to the International 10–20 system (Fp1, Fp2, F7, F3, Fz, F4, F8, FC5, FC1, FC2, FC6, T7, A1, C3, Cz, C4, T8, A2, CP5, CP1, CP2, CP6, P7, P3, Pz, P4, P8, PO3, PO4, O1, Oz, O2), and impedance was reduced to < 5 kΩ prior to data acquisition.

Shooting performance metrics was automatically logged by the MSH‐1 Light‐Weapons Training System (Beijing Zhongke Jiecheng, China). For each shot, the system extracted seven metrics: SCORE (shooting rings; 0 for a miss, 5.0–10.0 for a hit; higher is better); SX and SY (horizontal and vertical deviation of the aiming point; lower is better); COG (mean distance of the aiming trajectory from the bullseye; smaller is better); ATI (aiming time); RTV (trigger‐pull smoothness; lower values indicate less sight wobble); and TIRE (trigger timing and responsiveness; 1 = poor, 3 = optimal).

Upon completion of each 60‐shot block, participants completed an eight‐item self‐report questionnaire addressing perceived difficulty, stress, effort, participation, anxiety, fatigue, expectation (discrepancy between actual and expected performance), and self‐assessment of shooting ability. All items were rated on a 7‐point Likert scale. The anxiety item was adapted from the State‐Trait Anxiety Inventory (Michalos 2014), the stress item referenced the Perceived Stress Scale (Cohen et al. 1983), and the difficulty, effort, and expectation items were drawn from the NASA Task Load Index (≪Development of NASA‐TLX (task load index): Results of empirical and theoretical research≫, 1988). The remaining three items were newly developed based on shooting‐task characteristics to provide a more comprehensive assessment of subjective states during the task. The full questionnaire is provided in Supplementary Material 16.

A priori power analysis (G*Power 3.1; Faul et al. 2007) indicated that a repeated‐measures ANOVA (effect size = 0.25, power = 0.80, alpha = 0.05) required a minimum of 28 participants; 35 shooters were therefore recruited. Two datasets were excluded owing to poor signal quality, two to missing channels, and one to physical discomfort during the task, leaving 30 participants for analysis.

### 2.2 Data Pre‐Processing

The EEG data were processed offline on the MATLAB R2023a platform. First, a 1–30 Hz band‐pass filter was applied using EEGLAB (Delorme and Makeig 2004). Epochs were segmented from −4 s to 1 s relative to the shot onset, as illustrated in Figure 1A. Bad segments were discarded, bad channels were interpolated, and independent component analysis (ICA) was used to remove eye‐movement artifacts. Finally, the data were re‐referenced to the common average. After processing, 1759 trials remained under the Hostage‐Rescue Condition, 1723 under the Long‐Range Condition, and 1718 under the Close‐Range Condition, yielding mean trial counts per participant of 59, 57, and 57, respectively.

This study collected three types of experimental data: EEG signals during the shooting preparation phase, shooting performance metrics, and self‐report measures. From the preprocessed EEG data, four categories of features were extracted: sensor‐level spectral features, source‐space spectral features, functional connectivity features, and graph‐theoretic features, as illustrated in Figure 1B–D.

(1) Sensor‐level spectral features. These features include relative band power and ERD/ERS characteristics. To reduce inter‐individual variability among the 30 participants, individual alpha frequency (IAF) was determined using the peak frequency method (Goljahani et al. 2012). Based on the IAF, the theta band was defined as IAF − 6 to IAF − 2 Hz, the alpha band as IAF − 2 to IAF + 3 Hz, and the beta band as IAF + 3 to IAF + 20 Hz. The EEG signals were bandpass‐filtered into these three frequency bands. EEG data from −3.5 to −0.5 s and 0 to 1 s relative to shot onset was divided into four 1‐s time windows (Win1–Win4). Relative band power was then computed for each window as the power within the theta, alpha, or beta band divided by the total power across these three bands. Additionally, EEG data from −4 s before to 1 s relative to shot onset was divided into five 1‐s time windows. The period from −4 to −3 s served as the baseline window (Win0). ERD/ERS features for Win1, Win2, Win3, and Win4 were calculated relative to Win0 using the following formula.

(1) $$ERD/ERS\left(\%\right)=\left(\frac{P_{\mathrm{window}}-P_{\mathrm{baseline}}}{P_{\mathrm{baseline}}}\right)\;\times 100\%.$$

In the formula, $P_{\mathrm{window}}$ denotes the average power of the EEG signal in the analysis time windows (Win1 to Win4), and $P_{\mathrm{baseline}}$ denotes the average power in the baseline time window (Win0).

(2) Source‐space spectral features. The theta (4–8 Hz), alpha (8–13 Hz), and beta (13–30 Hz) bands were defined for source‐level spectral analysis. Preprocessed EEG segments from −3.5 to −0.5 s relative to shot onset were used to perform single‐trial source reconstruction with standardized low‐resolution electromagnetic tomography (sLORETA) implemented in LORETA‐KEY (Fruhwirt et al. 2025; Fuchs et al. 2002; Jurcak et al. 2007). Absolute power was extracted for each band and averaged across trials, then relative spectral power was computed as the power in each band divided by the total power across theta, alpha, and beta combined.

(3) Functional connectivity features. Source localization was performed using the sLORETA method in Brainstorm (Tadel et al. 2011) on EEG data (–3.5 s to –0.5 s), mapping signals to 15,002 cortical vertices. Subsequently, these vertices were divided into 68 Regions of Interest (ROIs) based on the Desikan–Killiany neuroanatomical atlas (Desikan et al. 2006). The signals from all vertices within each ROI were averaged to obtain the ROI's average time series. The theta band was defined as 4–8 Hz, the alpha band as 8–13 Hz, and the beta band as 13–30 Hz. Time series from each ROI were bandpass‐filtered within these frequency bands, followed by Hilbert transform to construct the analytic signal and extract instantaneous phase information.

(2) $$z_{x}\left[n\right]=x\left[n\right]+i\cdot H\left\{x\left[n\right]\right\}=A_{x}\;\left[n\right]e^{i\theta_{x}\left[n\right]},$$

(3) $$z_{y}\left[n\right]=y\left[n\right]+i\cdot H\left\{y\left[n\right]\right\}=A_{y}\;\left[n\right]e^{i\theta_{y}\left[n\right]}.$$

In the formula, x[n] and y[n] represent signals from different ROIs, H{·} denotes the Hilbert transform, $z_{y}\left[n\right]$ is the analytic signal of y[n], $z_{x}\left[n\right]$ is the analytic signal of x[n], $A_{x}$ and $A_{y}$ denote the instantaneous amplitudes of x[n] and y[n] respectively, $\theta_{x}\left[n\right]$ and $\theta_{y}\left[n\right]$ denote their instantaneous phases, and n denotes the discrete time index.

Then calculate the PLV.

(4) $$\mathrm{PLV}\;=\left|\frac{1}{N}\sum_{n=0}^{N-1}e^{i\Delta \theta \left[n\right]}\right|=\left|\frac{1}{N}\sum_{n=0}^{N-1}e^{i\left(\theta_{x}\left[n\right]-\theta_{y}\left[n\right]\right)}\right|.$$

In the formula, Δθ[n] represents the instantaneous phase difference between the two signals. $\theta_{x}\left[n\right]$ and $\theta_{y}\left[n\right]$ denote the instantaneous phases of x[n] and y[n], respectively. n denotes the discrete time index.

(4) Graph‐theoretic features. Graph‐theoretic features were extracted using the GRETNA toolbox (Wang et al. 2015) and the functional connectivity matrix. To enable the assessment of small‐worldness (*σ*), the 68 × 68 fully connected matrix composed of 68 ROIs underwent sparsification via the proportional thresholding method. First, the sparsity range was set from 0.06 to 0.6 with increments of 0.01 to compute the Sigma for all subjects across all frequency bands and conditions. Second, at each sparsity level, the average *σ* was calculated for subjects sharing the same frequency band and condition. The sparsity level corresponding to the first instance where this average dropped below 1 from above 1 was defined as the sparsity upper bound for that frequency band and condition (Pang et al. 2020). Subsequently, within the updated sparsity range (0.06 to the upper limit), the random network was simulated 500 times to compute three global metrics: *σ*, clustering coefficient (CC), and global efficiency (GE), along with three nodal metrics for 68 ROIs: betweenness centrality (BC), nodal clustering coefficient (NCC), and nodal efficiency (NE) (Ismail and Karwowski 2020). Finally, the area under the curve (AUC) of all metrics across the sparsity range serves as the final feature for graph‐theoretic analysis, comprehensively reflecting their stability and representativeness under network topology variations.

### 2.3 Statistical Analysis

(1) Shooting performance metrics and self‐report measures were separately examined for differences among the three experimental conditions. The mean of each metric was computed for every participant under each condition. A one‐way repeated‐measures analysis of variance (ANOVA) with condition as the within‐subject factor was conducted on each metric to obtain *p*‐values, *F*‐values, and partial eta‐squared (*η* ^2^). Subsequently, paired‐samples *t*‐tests were used for pairwise contrasts, reporting *p* values and Cohen's *d*. False‐discovery‐rate (FDR) correction was applied to the resulting *p*‐values, and the significance level was set at *p* = 0.05. Metrics that showed a significant main effect of condition were followed up with Scheffé’s post hoc test.

(2) Sensor‐level spectral features were tested for differences among conditions and pre‐shot time windows. A 2 × 3 two‐way repeated‐measures ANOVA (Condition × Time Window) was conducted on relative spectral power and ERD/ERS. This yielded *p*‐values, *F*‐values, and partial *η* ^2^ for each metric. False‐discovery‐rate (FDR) correction was applied to the resulting *p*‐values, and the significance level was set at *p* = 0.05. Metrics that showed a significant main effect of condition were followed up with Scheffé’s post hoc test.

(3) Source‐space spectral features were examined for differences among conditions. Paired‐sample *t*‐tests were performed in LORETA‐KEY. Family‐wise‐error (FWE) correction was implemented via 5000 permutations, with the significance level set at *p* = 0.05.

(4) Functional‐connectivity differences among conditions were assessed with paired‐sample *t*‐tests, yielding *p*‐values and Cohen's *d*. FDR correction was applied, and the significance level was set at *p* = 0.01.

(5) Graph‐theoretic features were compared among conditions using paired‐sample *t*‐tests, yielding *p*‐values and Cohen's *d*. FDR correction was applied, with the significance level set at *p* = 0.01.

(6) Additionally, Spearman's rank correlation was used to examine the relationship between EEG features and both shooting performance and subjective experience. Specifically, shooting performance metrics were correlated with electrode‐level spectral power, connectivity strength, and graph metrics, and self‐report measures were correlated with connectivity strength and graph metrics. FDR correction was applied, with the significance level set at *p* = 0.05. All correlation outcomes are reported in Supplementary Material 15.

## 3 Results

### 3.1 Shooting Performance Indicators and Self‐Report Measures

**FIGURE 2:**
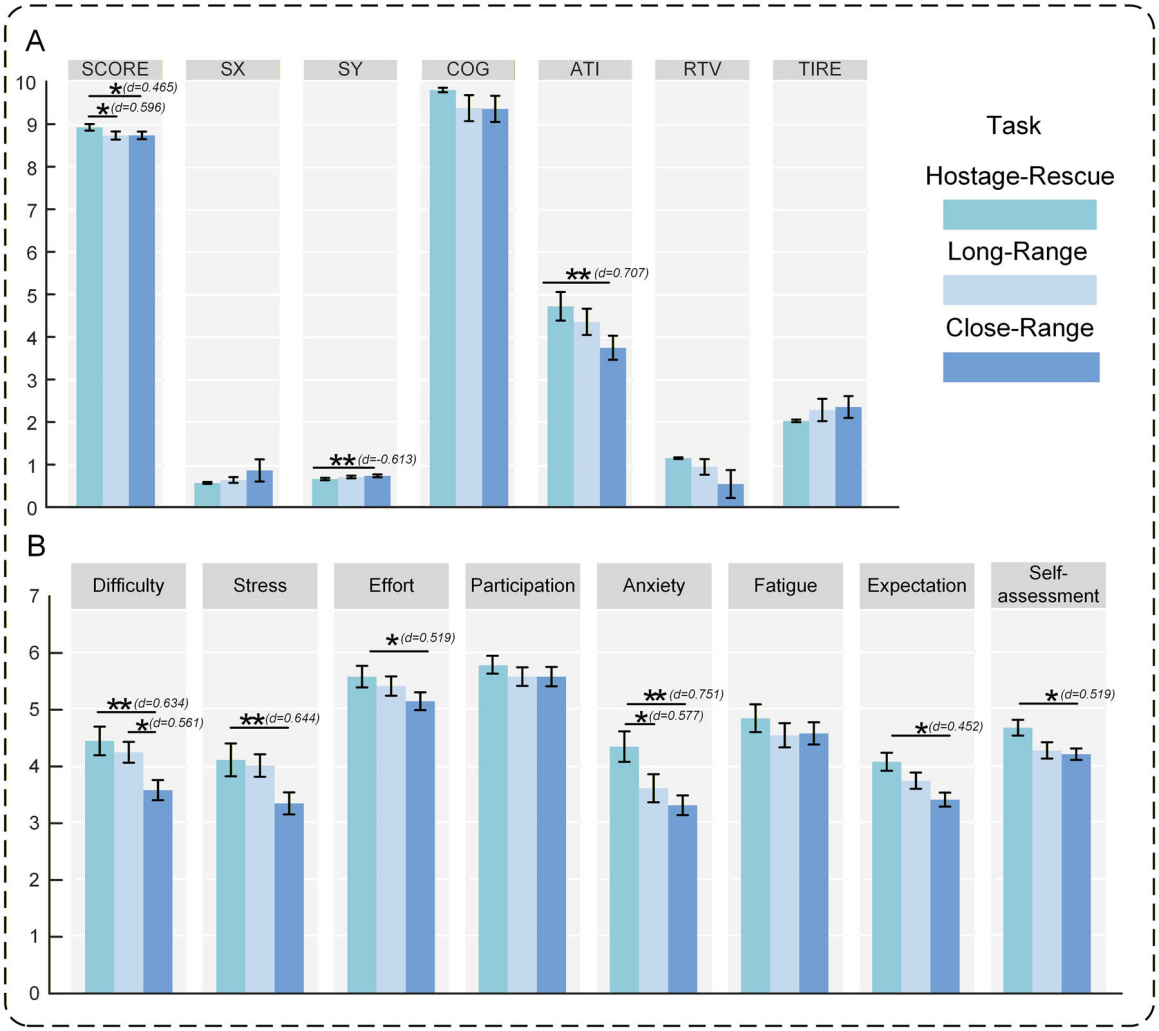
Statistical‐test results for shooting performance metrics and self‐report measures: (A) means, standard errors, and between‐condition comparisons for shooting performance metrics; (B) means, standard errors, and between‐condition comparisons for self‐report measures. *p < 0.05, **p < 0.01. *d* = Cohen's *d* (effect size for paired *t*‐tests).

The statistical analysis results for shooting‐performance metrics and self‐report measures are presented in Figure 2. As illustrated in Figure 2A, the SCORE in the Hostage‐Rescue Condition was significantly higher than in the Long‐Range and Close‐Range conditions, whereas SY was significantly lower than in the Close‐Range and Long‐Range conditions. Additionally, the ATI in the Hostage‐Rescue Condition and Long‐Range Condition was significantly higher than in the Close‐Range Condition. The statistical analysis results for self‐report measures are displayed in Figure 2B. Under the Hostage‐Rescue Condition, scores for Difficulty, Stress, Effort, Anxiety, Expectation, and Self‐assessment were significantly higher than those under the Close‐Range Condition. Under the Long‐Range Condition, Difficulty was significantly higher than those under the Close‐Range Condition. Furthermore, Anxiety scores under the Hostage‐Rescue Condition were significantly higher than those under the Long‐Range Condition.

Complete one‐way repeated‐measures ANOVA outcomes (*p*‐values, *F*‐values, partial *η* ^2^) are provided in Supplementary Materials 1 and 2.

### 3.2 Sensor‐Level Spectral Features

**FIGURE 3:**
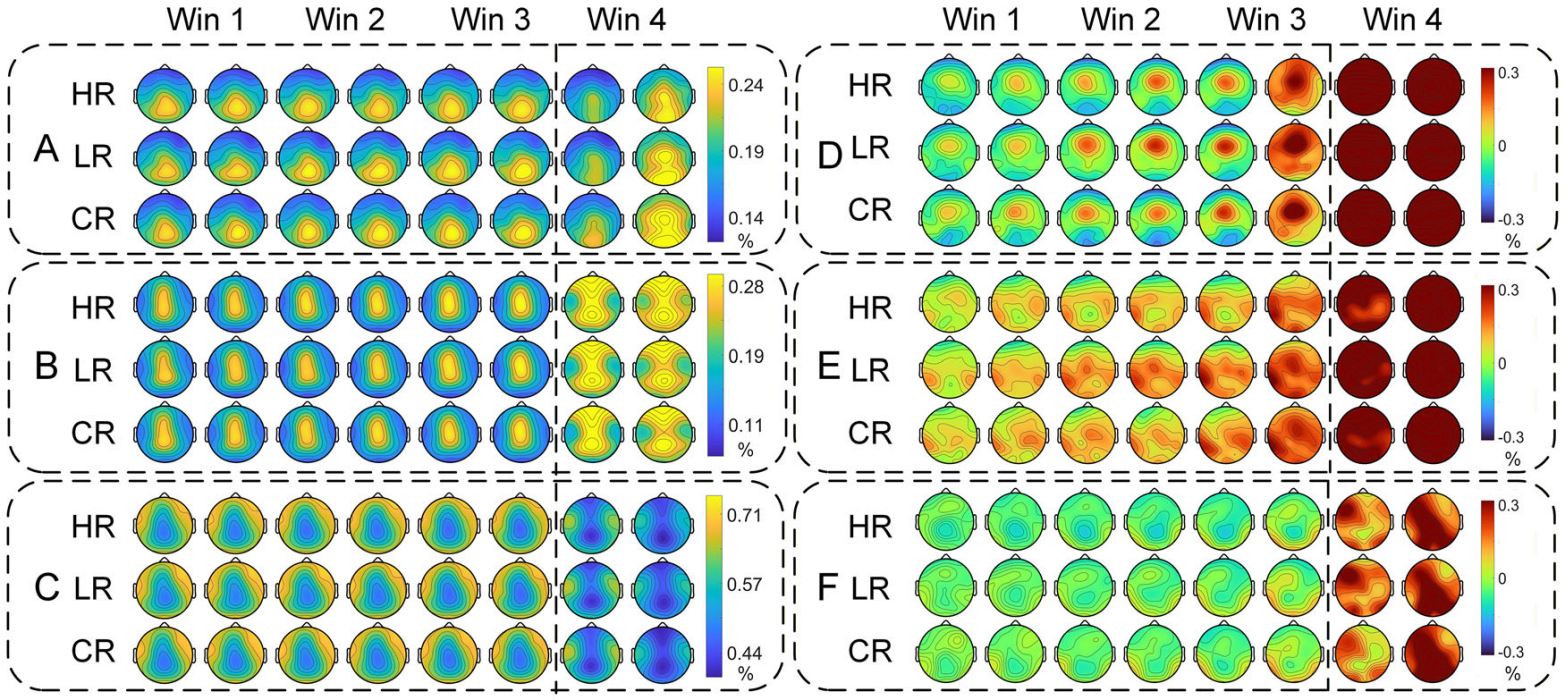
Sensor‐level spectral features across frequency bands and experimental conditions. (A–C) Relative band power for theta, alpha, and beta bands, respectively. (D–F) ERD/ERS for theta, alpha, and beta bands, respectively. Ch = Hostage‐Rescue, LR = Long‐Range, CR = Close‐Range. Win1–Win3 = pre‐trigger epochs (each partitioned into two consecutive sub‐windows to depict temporal trends); Win4 = post‐trigger epoch. Significance threshold *p* = 0.05 with multiple‐comparison correction.

Sensor‐level spectral features are displayed in Figure 3; relative spectral power is presented in Figure 3A–C. In the theta band, relative spectral power before shot onset was initially concentrated over the central scalp in all three conditions, declined across the subsequent time window, and concurrently increased at Fz, FC1, FC2, and Cz. Specifically, under the Hostage‐Rescue Condition, power at Fp2, F7, and O1 was significantly lower than under the Close‐Range Condition, whereas power at Cz, C3, T7, and CP1 was significantly higher than under the Long‐Range Condition; under the Long‐Range Condition, power at F7, T7, PO4, and O1 was significantly lower than under the Close‐Range Condition. In the alpha band, power was maximal over parietal regions and increased across time windows; after shot onset, whole‐brain power increased significantly. Specifically, the Hostage‐Rescue Condition showed higher power at F4 and FC6 than the Close‐Range Condition and higher power at F4, F8, FC6, and T7 than the Long‐Range Condition. In the beta band, power before shot onset was maximal over frontal, occipital, and bilateral temporal regions; after shot onset, whole‐brain power decreased significantly. Before shot onset, the Hostage‐Rescue Condition exhibited lower power than the Long‐Range Condition at F4, Cz, T7, T8, and CP1 and lower power than the Close‐Range Condition at FC6.

ERD/ERS are shown in Figure 3D–F. In the theta band, ERS was centered over FC1, whereas ERD dominated the remaining channels; across time windows, ERD at O1, O2, and Oz initially intensified and then diminished, while ERD at other sites transitioned to ERS that strengthened progressively. After shot onset, ERS was evident globally. In the alpha band, ERD was localized to FP1 and FP2, whereas ERS dominated the remaining channels; ERS increased globally across time windows, and after shot onset global ERS was observed.

In the second window, the Long‐Range Condition exhibited a more pronounced and rapid ERS increase over central, left parietal, and temporal sites, whereas the Hostage‐Rescuecondition showed a smaller, slower ERS increase at CP1. In the beta band, the first window was dominated by ERD; ERS subsequently emerged at P7, P8, O1, O2, and Oz, whereas ERD persisted elsewhere, and after shot onset global ERS was observed.

Two‐way repeated‐measures ANOVA outcomes (*p*‐values, *F*‐values, partial *η* ^2^) for relative power and ERD/ERS are provided in Supplementary Materials 3–6. Detailed sensor‐level spectral features correlations with both imaging‐performance metrics and self‐reported measures are provided in Figures S1 and S2.

### 3.3 Source‐Space Spectral Features

**FIGURE 4:**
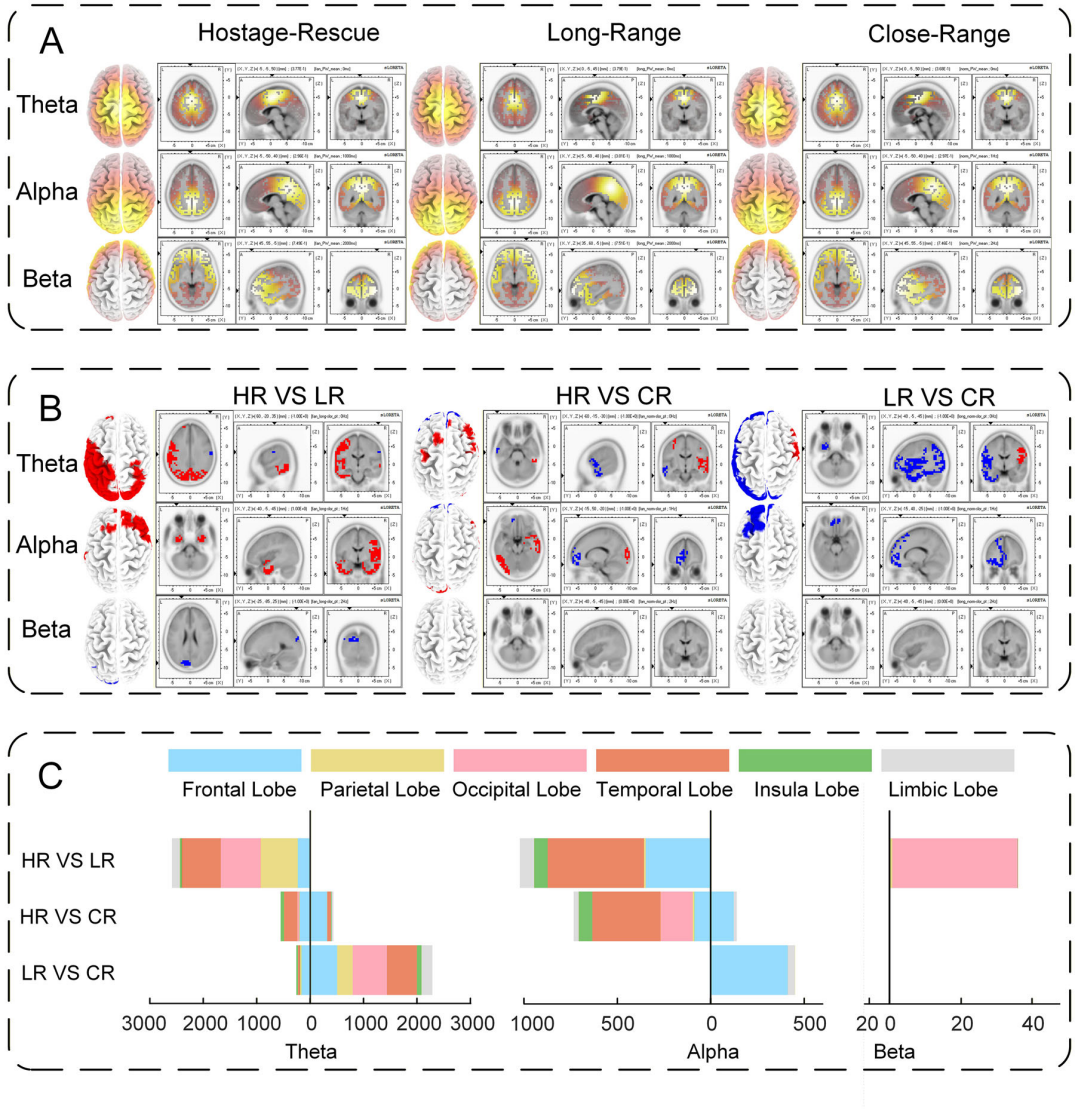
Source‐space spectral features and statistical analysis. (A) Relative spectral power distribution. (B, C) Statistical‐test results for relative spectral power. In B, red indicates the former is significantly greater than the latter, while blue indicates the former is significantly less than the latter. In C, the horizontal axis represents the number of voxels exhibiting significant differences, with different colors denoting distinct brain lobes; the vertical axis indicates statistical tests for different conditions: HR denotes the Hostage‐Rescue Condition, LR the Long‐Range Condition, and CR the Close‐Range Condition. Positions left of the vertical axis indicate the former is significantly greater than the latter, whereas positions right indicate the latter is significantly greater than the former. All statistical tests were conducted at a significance level of 0.05 and underwent multiple‐comparison correction.

Source‐space spectral features are displayed in Figure 4A, where the spatial distribution of EEG power is shown to vary across frequency bands: theta‐band relative power is concentrated primarily in the parietal lobe, alpha‐band relative power is concentrated primarily in the parietal and occipital lobes, and beta‐band relative power is centered mainly in the frontal and temporal lobes. As shown in Figure 4B and C, the theta band contained the largest number of significantly different voxels across conditions, followed by the alpha and beta bands. Within the theta band, relative spectral power in the vast majority of voxels was significantly higher under the Hostage‐Rescue Condition than under the Long‐Range Condition, and likewise higher under the Close‐Range Condition than under the Long‐Range Condition. In the alpha band, the majority of voxels exhibited significantly greater relative power under the Hostage‐Rescue Condition relative to both the Long‐Range and Close‐Range conditions, whereas the Close‐Range Condition showed significantly greater power than the Long‐Range Condition. In the beta band, most voxels displayed significantly higher power under the Close‐Range and Long‐Range conditions than under the Hostage‐Rescue Condition.

Detailed statistical results are provided in Supplementary Material 7.

### 3.4 Functional Connectivity Features

**FIGURE 5:**
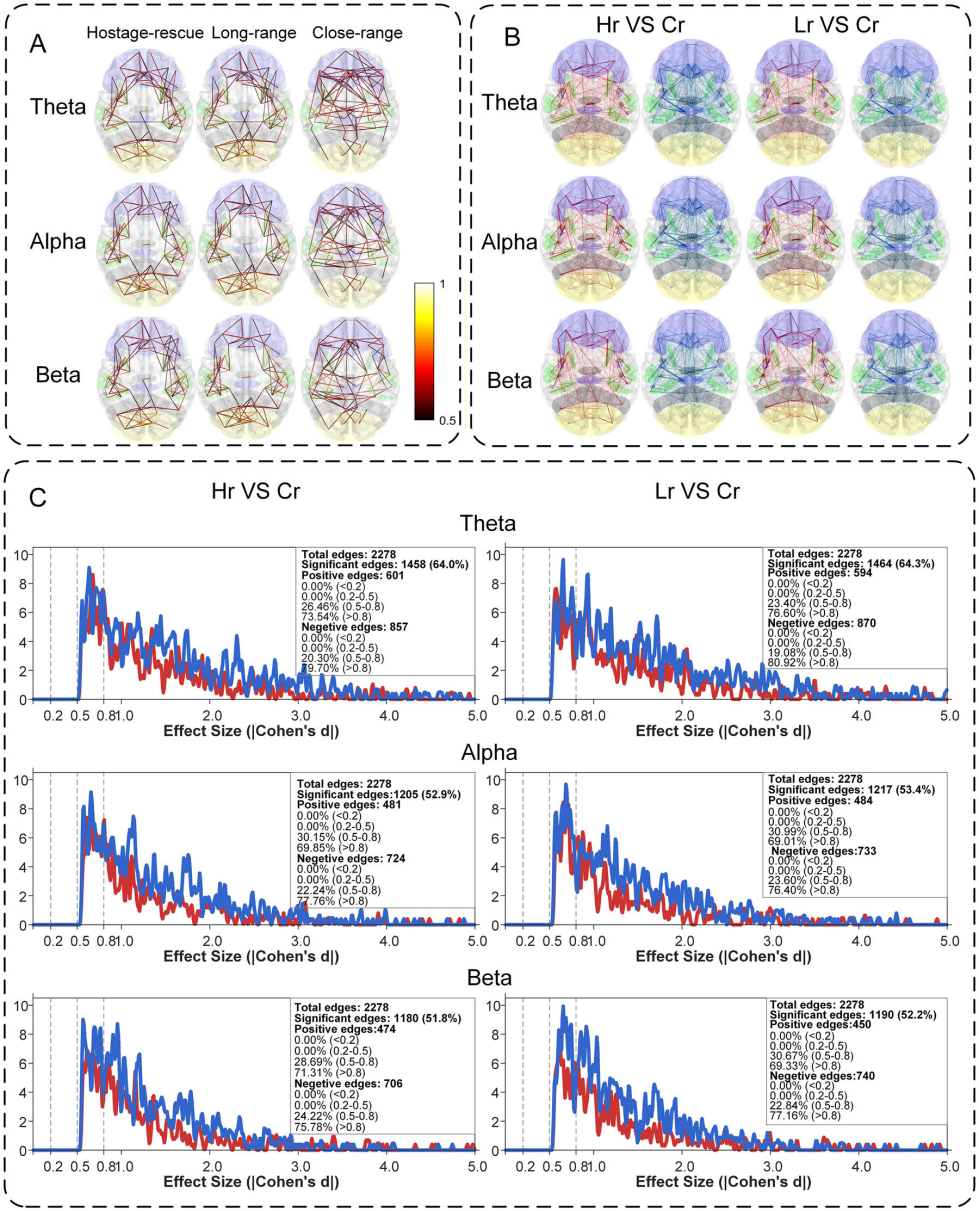
Functional connectivity features and statistical test results. (A) Functional connectivity map displaying only the top 5 % of connections with the highest values. (B) Statistical test results map where Ch denotes the Hostage‐Rescue Condition, LR the Long‐Range Condition, and CR the Close‐Range Condition; red connections indicate that the former is greater than the latter, whereas blue connections indicate the opposite. The darker and thicker the lines in the figure, the larger the absolute effect size. Node colors represent brain lobes: purple, frontal; green, temporal; gray, parietal; yellow, occipital. (C) Distribution of absolute effect sizes for significantly different functional connections. Red connections indicate the former is greater than the latter, blue connections indicate the latter is greater than the former. The significance level is set at 0.01 and all results have undergone multiple‐comparison correction.

Functional‐connectivity distributions and statistical outcomes are displayed in Figure 5. Figure 5A illustrates that, within the top 5% PLV connections across all frequency bands, connectivity patterns under the Hostage‐Rescue and Long‐Range conditions were similar to each other but differed markedly from those under the Close‐Range Condition. Connectivity between left and right occipital regions and within the left occipital lobe was higher under the Hostage‐Rescue and Long‐Range conditions than under the Close‐Range Condition, whereas inter‐frontal connectivity was higher under the Close‐Range Condition than under the Hostage‐Rescue and Long‐Range conditions.

Statistical outcomes are summarized in Figure 5B. Connectivity differences relative to the Close‐Range Condition were comparable for the Hostage‐Rescue and Long‐Range conditions, with no significant difference between the latter two. Enhanced connectivity under the Hostage‐Rescue and Long‐Range conditions was dispersed across frontal, occipital, temporal, and parietal regions, especially in the beta band; reduced connectivity under the same two conditions was concentrated within ipsilateral frontal, frontal‐temporal, and frontal‐parietal pathways, particularly in the theta band.

Pairwise *t*‐test results (*p*‐values and Cohen's *d*) are provided in Supplementary Materials 8 and 9. ROI‐to‐lobe mappings for the 68 regions are given in Supplementary Material 10. Correlations between functional connectivity and both shooting performance metrics and self‐report measures are displayed in Figure S3.

### 3.5 Graph‐Theoretic Features

**FIGURE 6:**
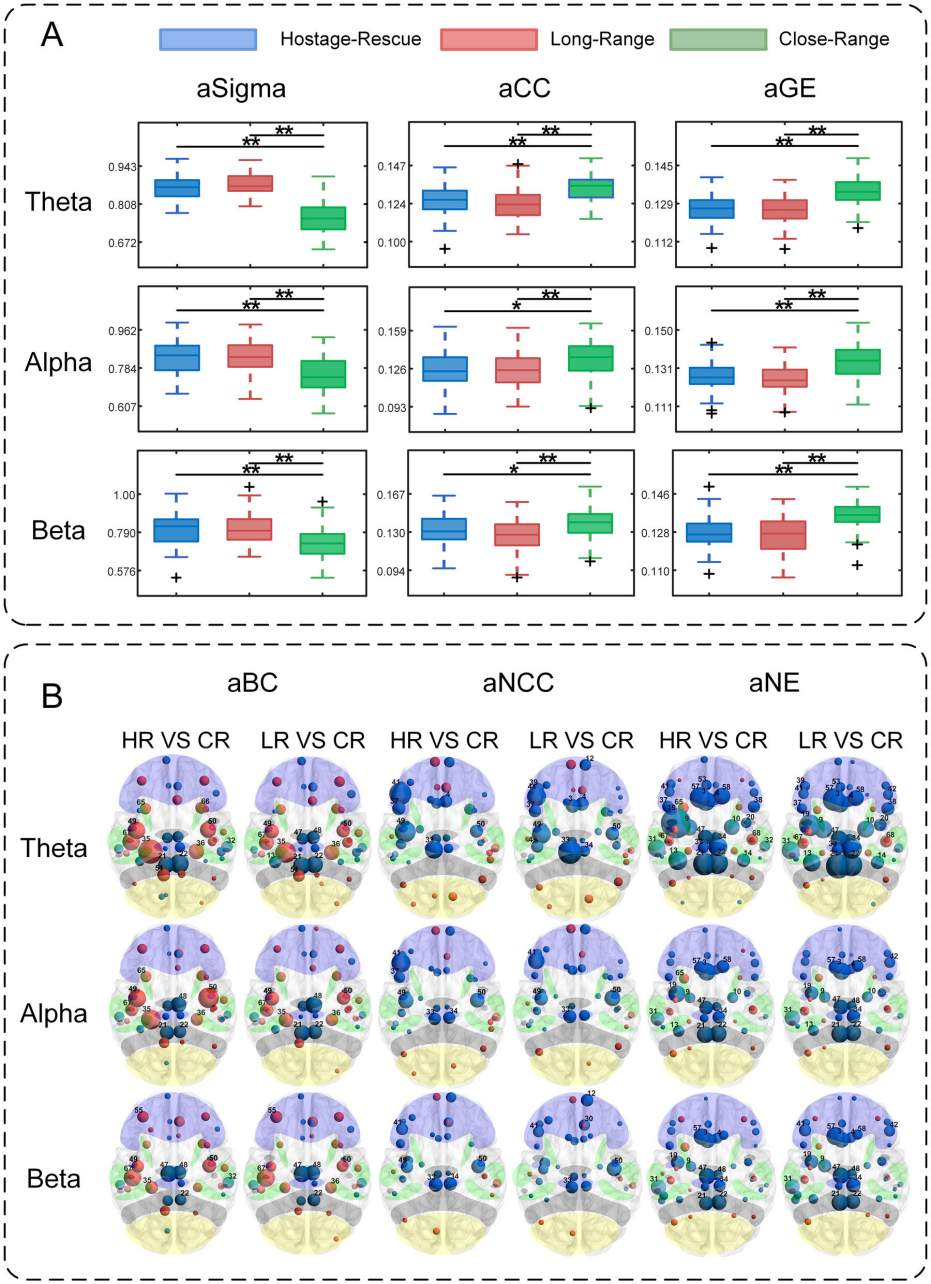
Graph‐theory features and statistical test results. (A) Statistical test results for global metrics (* indicates *p* < 0.01). (B) Statistical test results for global metrics. Different colors represent different brain lobes: purple indicates the frontal lobe, green indicates the temporal lobe, gray indicates the parietal lobe, and yellow indicates the occipital lobe. Nodes represent ROIs. Red indicates that the former value is greater than the latter, blue indicates that the latter is greater than the former, and node size represents the magnitude of the absolute effect size. Ch denotes the Hostage‐Rescue Condition, LR denotes the Long‐Range Condition, and CR denotes the Close‐Range Condition. The statistical test significance level was set at 0.01 and underwent multiple‐comparison correction.

Statistical results for graph‐theoretic metrics are shown in Figure 6. Figure 6A indicates that *σ* was significantly higher under the Hostage‐Rescue and Long‐Range conditions than under the Close‐Range condition across all frequency bands, whereas CC and GE were significantly lower under the same two conditions than under the Close‐Range Condition.

Nodal‐metric outcomes are summarized in Figure 6B: across all frequency bands, differences relative to the Close‐Range Condition were comparable for the Hostage‐Rescue and Long‐Range conditions, with no significant difference found between the latter two. BC was significantly higher in temporal ROIs and lower in parietal ROIs under the Hostage‐Rescue and Long‐Range conditions than under the Close‐Range Condition. NCCs were significantly higher in occipital ROIs and significantly lower in left frontal and temporal ROIs under the Hostage‐Rescue and Long‐Range conditions than under the Close‐Range Condition. Significant hemispheric asymmetry was observed in the frontal and temporal regions. NE was significantly lower in frontal and parietal ROIs under the Hostage‐Rescue and Long‐Range conditions than under the Close‐Range Condition.

Paired‐sample *t*‐test outcomes (*p*‐values and Cohen's *d*) are provided in Supplementary Materials 11–14. Correlations between graph‐theoretical metrics and both shooting performance metrics and self‐report measures are displayed in Table S1.

## 4 Discussion

### 4.1 Sensor‐Level Spectral Features

From a temporal‐dynamics perspective, as the trigger pull approached, most channels exhibited beta ERD that shifted to beta ERS after firing. This aligns with prior reports of beta ERD over the central–parietal region before stimulus or cue onset, followed by beta ERS after motor execution (Pastotter et al. 2008), reflecting pre‐firing activation readiness and post‐firing relative inhibition.

Across shooting conditions, the Hostage‐Rescue Condition exhibited significantly lower relative beta‐band power over frontal, central, and temporal channels than the Long‐Range Condition. Previous work links endogenous beta oscillations (15–30 Hz) to visual‐perceptual integration (Aissani et al. 2014), decision‐making (Wimmer et al. 2016), and other cognitive functions (Engel and Fries 2010) and indicates that beta activity increases during states of focused attention, active thought, and alertness (Perlis et al. 2001). Nevertheless, excessive beta activity is associated with heightened anxiety and arousal, impeding relaxation (Rajan et al. 2024); it is possible that the Long‐Range Condition was accompanied by greater arousal and anxiety than the Hostage‐Rescue Condition.

Comparison of spectral features across frequency bands revealed that the beta band exhibited the greatest number of significant differences in ERD/ERS across conditions. This finding is likely due to the association between beta activity and states of anxiety and arousal (Bayazit and Ungur 2018; Roxburgh et al. 2023), suggesting that beta‐band dynamics serve as the sensitive marker of stress and arousal differences among the shooting conditions.

Correlation analysis results indicate that, compared to the Hostage‐Rescue and Close‐Range conditions, the relative power of the alpha band exhibits the strongest negative correlation with RTV under the Long‐Range Condition. This negative correlation is significant across multiple channels in the frontal, central, parietal, and occipital lobes. This may indicate that, during the long‐range shooting condition that demands high precision, the brain enhances alpha‐band activity to achieve better attentional control and motor inhibition, thereby improving trigger‐control smoothness.

### 4.2 Source‐Space Spectral Features

In the statistical analysis of source‐space spectral features, the theta band exhibited the highest number of voxels showing significant differences across conditions, potentially reflecting the critical role of theta oscillations in cognitive and emotional regulation (Zouaoui et al. 2023). Specifically, relative theta power in the occipital, temporal, frontal, and parietal lobes was significantly higher under the Hostage‐Rescue and Close‐Range conditions than under the Long‐Range Condition. Given that theta oscillations serve as a key neural mechanism for cognitive‐reappraisal strategies that enhance emotional responses to negative stimuli (Zouaoui et al. 2023), this finding may suggest that, under the Hostage‐Rescue and Close‐Range conditions, participants effectively mobilized greater cognitive‐reappraisal resources, thereby amplifying emotional processing of negative information to facilitate risk assessment and decision‐making.

Additionally, relative theta and alpha power was significantly higher across the insula and limbic lobes in the Hostage‐Rescue Condition than in the Long‐Range Condition. Similarly, relative theta and alpha power in the anterior and posterior cingulate cortices of the limbic system was significantly higher during the Close‐Range Condition than during the Long‐Range Condition. Previous research indicates that the insula integrates emotion‐related cues and that activation in the insula and anterior cingulate cortex is typically associated with intrinsic emotion processing (Zhou et al. 2020). This finding may suggest that, under the Hostage‐Rescue and Close‐Range conditions, subjects demonstrated superior emotional monitoring and self‐regulation capabilities compared with the Long‐Range Condition, leading to enhanced theta activity in these emotion‐processing brain regions.

### 4.3 Functional Connectivity Features

Functional connectivity under the Hostage‐Rescue and Long‐Range conditions was significantly greater than under the Close‐Range Condition, exhibiting greater dispersion and concentrating within individual lobes. Conversely, connections that were significantly weaker in the Hostage‐Rescue and Long‐Range conditions than in the Close‐Range Condition were more concentrated, primarily between the frontal lobe and other lobes. The frontal lobe—particularly the prefrontal cortex—plays a crucial role in integrating information from multiple association cortices and supports higher‐order cognitive functions such as attentional control, working memory, and behavioral inhibition through top‐down inhibitory mechanisms (Zhou et al. 2020). This may suggest that, in high‐risk scenarios (e.g., hostage situations) or tasks demanding high precision (e.g., long‐range shooting), limited cognitive resources prompt the brain to activate an adaptive resource‐conservation and efficiency‐optimization mechanism (Cheng et al. 2025). This mechanism temporarily weakens cross‐regional integration networks centered on the frontal lobe to reduce cognitive‐control resource expenditure, while enhancing information‐processing efficiency in localized brain regions to better address immediate perceptual and response demands.

Additionally, this study observed weakened functional connectivity between the frontal lobe and other lobes in skilled shooters during the Hostage‐Rescue and Long‐Range conditions. This pattern resembles the “frontal‐amygdala dysregulation” reported in patients with post‐traumatic stress disorder (PTSD). Early research indicates that PTSD is associated with an impaired capacity of the ventromedial prefrontal cortex to regulate amygdala fear responses (Vanuk 2022). Inhibitory interactions between the ventromedial prefrontal cortex and the amygdala are crucial for extinguishing conditioned fear responses, and dysfunction in this regulatory circuit contributes to persistent fear in PTSD (Åhs 2015). Furthermore, the inferior fronto‐occipital fasciculus might connect the frontal lobe and occipital lobe and modulate anxiety responses to environment stimulus (Liao 2014). Music therapy has been demonstrated to alleviate anxiety, potentially through enhancing functional connectivity between the frontal and occipital lobes (Misrani 2024). Therefore, the functional connectivity alterations observed in this study may result from the brain temporarily weakening top‐down prefrontal regulation during acute stress in high‐pressure, high‐risk task contexts. While this neuroadaptive mechanism facilitates rapid behavioral responses in urgent situations, prolonged or repeated exposure to similar high‐pressure environments may increase the risk of emotional dysregulation and stress‐related pathologies.

Correlation analysis results indicate that in the Long‐Range Condition, both the functional connectivity of the frontal‐parietal theta band and the frontal‐occipital beta band showed significant negative correlations with RTV. Since RTV reflects the severity of trigger‐pull tremor, this suggests that enhanced frontal‐occipital and frontal‐parietal connectivity under the Long‐Range Condition may promote action stability during trigger pull. In the Hostage‐Rescue Condition, the functional connectivity in the frontal‐parietal theta band showed a significant negative correlation with TIRE. Since TIRE measures the ability to trigger decisively and accurately at the optimal moment, reflecting sensorimotor integration and decision efficiency, this may suggest that enhanced frontal‐parietal connectivity during Hostage‐Rescue Condition may impair trigger‐timing control. This likely stems from the high‐risk nature of such scenarios—excessive cognitive monitoring may instead interfere with fluid motor execution. Compared with the Long‐Range and Close‐Range conditions, fatigue under the Hostage‐Rescue Condition was significantly positively correlated with frontal–parietal, frontal–occipital and frontal–frontal functional connectivity in the theta band, possibly because increased task load and psychological stress exhaust neural resources during sustained attention and heightened vigilance, leading to elevated subjective fatigue.

### 4.4 Graph‐Theoretic Features

This study found that the *σ* of the Hostage‐Rescue and Long‐Range conditions were significantly greater than those of the Close‐Range Condition, whereas the CC and GE were significantly lower than in the Close‐Range Condition. Previous research has shown that stress exposure affects the structure and function of brain connectivity (McEwen and Gianaros 2011). Experiments involving sleep deprivation—a physiological stressor—revealed a significant decline in GE during fatigue (Qi et al. 2021), and under psychosocial‐stress conditions GE was also reduced relative to control conditions (Wheelock et al. 2018). These results align with the reduced GE observed in the Hostage‐Rescue and Long‐Range conditions. Concurrently, prior work indicates that environmental stress correlates with reduced modularity in resting‐state brain networks, suggesting denser connections between modules and sparser connections within modules—a pattern thought to reflect diminished functional differentiation among modules (Jeong et al. 2024). Under intense urinary urgency (physiological stress), both the CC and NE were significantly decreased compared with the empty‐bladder state (Pang et al. 2020), mirroring the reduced CC observed in the Hostage‐Rescue and Long‐Range conditions.

Our study reveals that the occipital lobe exhibits significantly higher NCC in the Hostage‐Rescue and Long‐Range conditions than in the Close‐Range Condition, reflecting tighter local functional connectivity and enhanced information‐integration efficiency in the occipital lobe during the former states. Given the occipital lobe's involvement not only in primary visual processing but also in multisensory integration (Esteves et al. 2025), these findings demonstrate adaptive strategies for functional reorganization and resource reallocation within the brain network. Additionally, significant results exhibited asymmetry. Under the Hostage‐Rescue and Long‐Range conditions, the NCC in most ROIs of the left frontal and temporal lobes were significantly lower than under the Close‐Range Condition. The left hemisphere plays a central role in controlling motor speech, basic syntactic skills, and semantic processing (Kasselimis et al. 2018), and the left temporal lobe is a core component of the language system (Heyer et al. 2022); left frontal regions are crucial for audiovisual integration and language processing (Corrivetti et al. 2019). These observations may suggest that when cognitive load or processing demands increase, neural‐resource conservation and reallocation are achieved by reducing the NCC of local networks less relevant to the current core task, thereby maintaining the operational economy of brain networks. The findings also support the notion of “bias” in network‐resource allocation under stress, whereby stress may preferentially activate certain modules while suppressing others to optimize the stress response (Reinelt et al. 2019; Zhang et al. 2020). The Hostage‐Rescue and Long‐Range conditions exhibited significantly lower node‐efficiency metrics across most ROIs in the frontal and parietal lobes compared with Close‐Range Condition. Given that frontal and parietal lobes are closely associated with emotional processing (Cao 2020), this suggests a greater demand for emotional suppression and cognitive regulation under these two conditions. This additional cognitive load impairs the efficiency of information integration between these regions and other brain areas, leading to reduced nodal efficiency in the frontal and parietal lobes and ultimately diminishing global efficiency.

Within the correlation results, difficulty was significantly positively correlated with the clustering coefficient and global efficiency of the Hostage‐Rescue Condition in both the alpha and theta bands, possibly because participants perceived greater difficulty, the brain exhibited enhanced functional integration and local information‐processing capacity, reflecting a neural strategy that recruits more efficient network organization to cope with high‐demand, high‐risk situations.

### 4.5 Limitations and Future Directions

Previous simulation and empirical studies have indicated that, under conventional scalp‐coverage conditions, 32‐channel EEG systems are prone to introducing “spatial aliasing” effects during source imaging, potentially leading to radial positioning errors of 1–2 cm for dipoles (Song et al. 2015). Because the present study used a 32‐channel configuration for source localization, functional‐connectivity analysis, and graph‐theoretic examinations of brain‐network topology, such positioning biases are unavoidable. To mitigate their impact, we averaged source‐localization outcomes across all trials (5040 trials in total, encompassing all data from 30 subjects under the three experimental conditions). We hypothesize that increasing the number of trials entering the source computation may partially reduce random errors arising from fluctuations in dipole orientation and intensity. However, although this study effectively reduced the impact of random errors on population‐level results through extensive trial averaging, this strategy is difficult to replicate in individualized clinical diagnosis. In clinical practice, the available data for individual patients are typically limited. Moreover, graph‐theory metrics are highly sensitive to source‐localization accuracy, which affects the construction of functional‐connectivity matrices and the subsequent interpretation of network‐topology features. Therefore, while the current 32‐channel EEG‐based analytical framework is suitable for exploring neural mechanisms at the population level, its reliability requires careful evaluation when extended to individualized diagnosis or precision‐medicine scenarios. One study indicates that the most significant improvement occurs when the number of electrodes is increased from 32 to 64, with further increases yielding less pronounced effects. High‐density EEG with 64 or more electrodes, combined with sLORETA and independent component analysis algorithms, demonstrates high accuracy (Ahmad and Barkana 2025). Consequently, future work should prioritize validating the stability of high‐density EEG (e.g., 64‐channel or higher) in individual source imaging and graph‐theory analysis to support clinical translation.

It should be noted that, owing to practical recruitment constraints, the composition of military‐academy student cohorts, and their shooting‐training backgrounds, all participants were healthy young men. Consequently, the findings primarily apply to highly trained young males and cannot be directly extrapolated to women, civilians, or clinical populations such as those with anxiety disorders or PTSD. Therefore, future studies should expand sample coverage to include women, non‐military individuals, and individuals with specific psychological or physiological disorders; such expansion would enable systematic examination of similarities and differences in stress responses and shooting performance across diverse populations, thereby enhancing the study's generalizability and clinical relevance.

Furthermore, the Hostage‐Rescue Condition used here may induce mild acute stress. All participants provided written informed consent and could withdraw at any time, ensuring that overall risk remained controllable. However, if similar high‐fidelity or high‐intensity stress paradigms are extended to clinical or non‐military populations in the future, ethical risks must be rigorously assessed. We recommend strengthening pre‐enrollment psychological screening, establishing stricter real‐time withdrawal mechanisms, and providing necessary psychological counseling and support after the experiment to minimize the potential for inducing distress or long‐term psychological discomfort.

Finally, biofeedback may offer a non‐invasive, effective psychophysiological intervention for mental disorders. The clinical efficacy of biofeedback has been studied across a range of conditions, including anxiety, depression, and schizophrenia. Biofeedback that targets maladaptive physiological responses may help patients recognize and modify problematic bodily symptoms that contribute to—or perpetuate—related psychological issues (Schoenberg and David 2014). Moreover, biofeedback‐assisted behavioral medicine not only effectively improves patients’ health and quality of life but also significantly reduces healthcare costs, making it a cost‐effective strategy worthy of broader implementation (Schneider 1987). Therefore, weakened frontal‐to‐other‐lobe connectivity, enhanced small‐world properties, reduced network‐average clustering coefficient, and decreased global efficiency may collectively serve as objective biomarkers for identifying stress or state anxiety under Hostage‐Rescue and Long‐Range conditions. These findings hold promise for future applications that enable healthcare professionals to conduct early identification or personalized stress‐recovery training for high‐stress occupational groups, such as military personnel and surgeons.

## 5 Conclusions

This study integrated sensor‐level spectral, source‐space spectral, functional‐connectivity and graph‐theoretic features derived from 32‐channel scalp EEG to quantify how high‐risk (Hostage‐Rescue) versus high‐precision (Long‐Range) shooting demands modulate cortical neuromodulatory mechanisms and shooting performance, yielding four main outcomes: (1) Perceived pressure rose significantly under both Hostage‐Rescue and Long‐Range conditions, yet shooting score and aiming time improved only in the Hostage‐Rescue Condition. (2) Multi‐band, multi‐regional differences emerged at both sensor and source levels. At the sensor level, spectral analysis revealed the greatest number of significant differences in beta‐band ERD/ERS across conditions, a finding potentially attributable to the well‐documented association between beta oscillations and anxiety/arousal levels. Source‐level analysis showed the theta band exhibiting the greatest number of significant voxels differences across conditions, possibly reflecting theta oscillations' critical role in cognitive and emotional regulation. (3) Shooting under the Hostage‐Rescue and Long‐Range conditions induces changes in brain connectivity patterns, reducing functional connectivity between the frontal lobe and other brain lobes—thereby decreasing the allocation of cognitive control resources—while simultaneously enhancing functional connectivity within brain lobes. (4) Shooting under the Hostage‐Rescue and Long‐Range conditions elicits systematic changes in network topology: in global metrics, *σ* increases whereas CC and GE decrease significantly; at nodal metrics, the same conditions increase the NCC in the occipital lobe while decreasing it in most left frontal and temporal lobe ROIs, reflecting adaptive neural resource allocation and efficiency optimization mechanisms within the brain.

## Author Contributions

All authors contributed to the study conception and design. Material preparation, data collection, and analysis were performed by Xinyu Shi and Anmin Gong. The first draft of the manuscript was written by Xinyu Shi and all authors commented on previous versions of the manuscript. All authors read and approved the final manuscript. The authors declare no competing interests.

## Funding

This study was partially funded by the National Natural Science Foundation of China (Grant Nos. 62006246; 82172058; 62376112; 81771926; 61763022) and the 73rd batch of the General Postdoctoral Science Foundation of China (2023M734315). In addition, there is Xi'an Science and Technology Association youth talent lifting plan (959202413100).

## Ethics Statement

This study was performed in accordance with the Declaration of Helsinki. This human study was approved by Ethics Review Committee of College of information Engineering, Engineering University of People's Armed Police Force. All adult participants provided written informed consent to participate in this study.

## Conflicts of Interest

The authors declare that the research was conducted in the absence of any commercial or financial relationships that could be construed as a potential conflict of interest.

## Supporting information



Supplementary Material: brb371261‐sup‐0001‐SuppMat.xlsx

Supplementary Material: brb371261‐sup‐0002‐SuppMat.xlsx

Supplementary Material: brb371261‐sup‐0003‐SuppMat.xlsx

Supplementary Material: brb371261‐sup‐0004‐SuppMat.xlsx

Supplementary Material: brb371261‐sup‐0005‐SuppMat.xlsx

Supplementary Material: brb371261‐sup‐0006‐SuppMat.xlsx

Supplementary Material: brb371261‐sup‐0007‐SuppMat.xlsx

Supplementary Material: brb371261‐sup‐0008‐SuppMat.xlsx

Supplementary Material: brb371261‐sup‐0009‐SuppMat.xlsx

Supplementary Material: brb371261‐sup‐0010‐SuppMat.xlsx

Supplementary Material: brb371261‐sup‐0011‐SuppMat.xlsx

Supplementary Material: brb371261‐sup‐0012‐SuppMat.xlsx

Supplementary Material: brb371261‐sup‐0013‐SuppMat.xlsx

Supplementary Material: brb371261‐sup‐0014‐SuppMat.xlsx

Supplementary Material: brb371261‐sup‐0015‐SuppMat.docx

Supplementary Material: brb371261‐sup‐0016‐SuppMat.docx

Supplementary Figure: brb371261‐sup‐0017‐FigureS1.jpg

Supplementary Figure: brb371261‐sup‐0018‐FigureS2.jpg

Supplementary Figure: brb371261‐sup‐0019‐FigureS3.jpg

## Data Availability

The data that support the findings of this study are available from the corresponding author, Gong, upon reasonable request.
