# Supplementary material for: Neural Mechanisms of Shooting Preparation Under High‐Risk and High‐Precision Tasks: A Multiscale EEG Study
Source: Brain Behav. 2026 Mar 9;16(3):e71261. doi: 10.1002/brb3.71261 (PMC12971187; doi:10.1002/brb3.71261)
Supplement: Supplementary file 15 — Supplementary Material: brb371261‐sup‐0015‐SuppMat.docx [file BRB3-16-e71261-s017.docx]

Supplementary Materials

I. Shooting Performance Metrics and Self-Report Measures

Statistical analyses of shooting performance metrics and self-report measures are provided in 01_Statistical_Analysis_of_Shooting_Performance_Metrics.xlsx and 02_Statistical_Analysis_of_Self-Report_Measures.xlsx.

II. Sensor-Level Spectral Features

Significant differences in relative spectral power across shooting conditions and time windows are reported in 03_PW_Cond_ANOVA.xlsx and 04_PW_Win_ANOVA.xlsx. Significant differences in ERD/ERS features across shooting conditions and time windows are reported in 05_ERDERS_Cond_ANOVA.xlsx and 06_ERDERS_Win_ANOVA.xlsx.

III. Source-Space Spectral Features

Significant differences in source-space activation across conditions are detailed in 07_sLORETA_Sources_Cond.xlsx. The file contains three worksheets presenting statistical results for the theta, alpha, and beta frequency bands.Abbreviations: HR = Hostage-Rescue Condition; Lr = Long-Range Condition; CR = Close-Range Condition. HR_LR(2788) indicates 2,788 voxels with significantly greater activation in the Hostage-Rescue Condition than in the Long-Range Condition. The worksheet lists the corresponding lobes, anatomical structures, Brodmann areas, and relative proportions of these voxels. Conversely, LR_HR(60) indicates 60 voxels with significantly greater activation in the Long-Range Condition than in the Hostage-Rescue Conditionn. Voxels accounting for <0.1 % of total volume are omitted.

IV. Functional Connectivity Features

Statistical results for functional connectivity across conditions are provided in 08_PLV_HR_CR_T_Test.xlsx and 09_PLV_LR_CR_T_Test.xlsx. Each file contains three worksheets (theta, alpha, beta) with abbreviations as defined above. The mapping between Desikan–Killiany atlas regions-of-interest (ROIs) and cerebral lobes is given in 10_DK_ROI_to_Lobes_Mapping.xlsx. The atlas comprises 68 ROIs (odd numbers = left hemisphere; even numbers = right hemisphere).

V. Graph-Theoretic Features

Statistical results for global metrics—small-worldness (σ), average clustering coefficient (CC), and global efficiency (Eg)—are presented in 11_Global_Metric_T_Test_Results.xlsx (three worksheets: theta, alpha, beta). Statistical results for nodal metrics—betweenness centrality (BC), nodal clustering coefficient (NCC), and nodal efficiency (NE)—are provided in 12_Nodal_Metric_aBC_T_Test_Results.xlsx, 13_Nodal_Metric_aNCC_T_Test_Results.xlsx, and 14_Nodal_Metric_aNE_T_Test_Results.xlsx, respectively (three worksheets each: theta, alpha, beta). Abbreviations are as defined above.

VI. Correlation Features

Fig.S 1 illustrates the correlations between spectral features and shooting-performance metrics. In the alpha band, the relative spectral power of the Long-Range Condition over the frontal, central, parietal and occipital regions was significantly negatively correlated with RTV across all three time windows. In the beta band, during the first window, the ERD/ERS of the Long-Range Condition over the central and frontal regions was significantly positively correlated with ATI; during the third window, the ERD/ERS of the Long-Range Condition over the frontal region was significantly positively correlated with SX, that over the occipital region was significantly positively correlated with ATI, that over the frontal and temporal regions was significantly positively correlated with TIRE, and that over the frontal and temporal regions was significantly negatively correlated with COG.


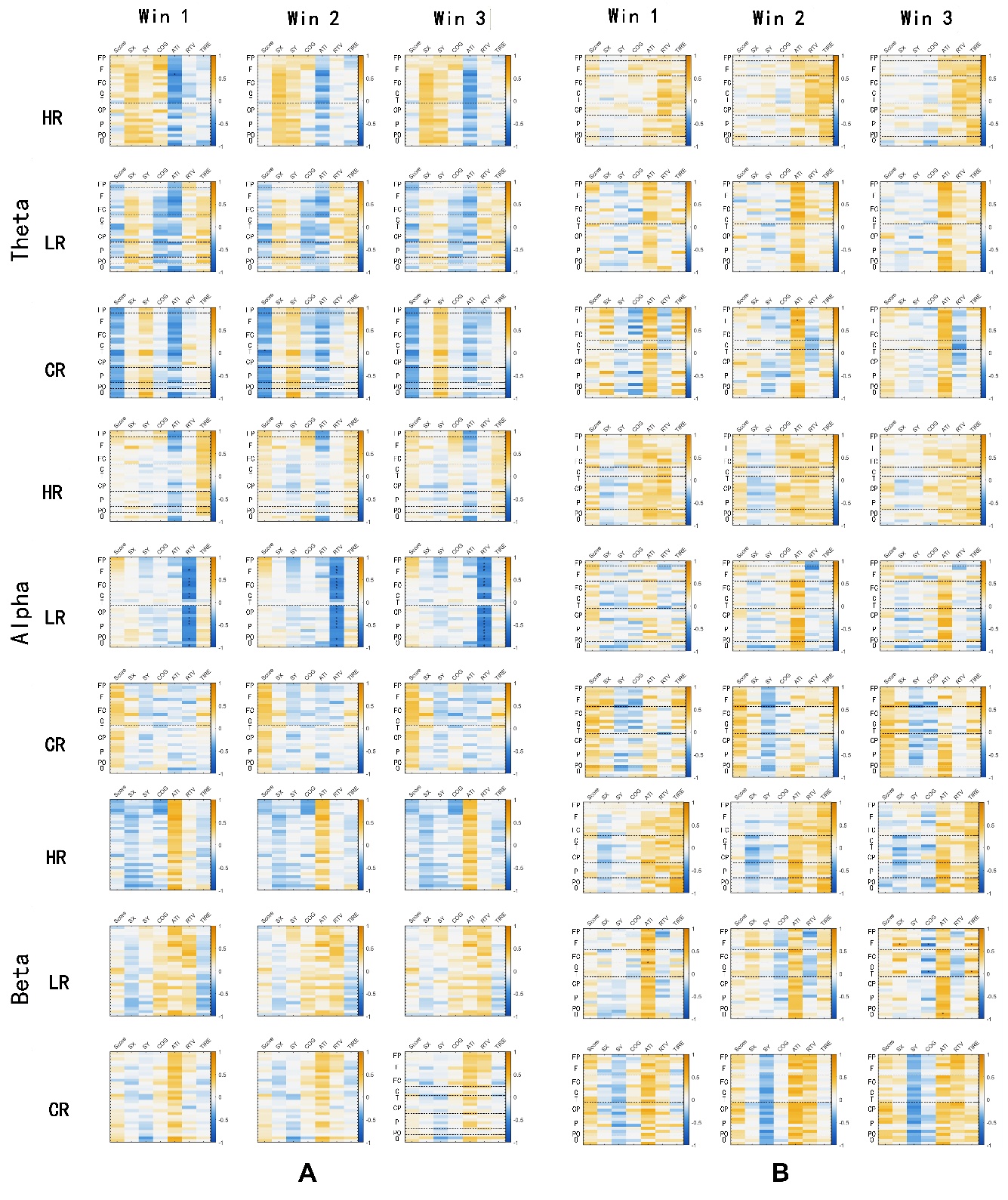


Fig.S 1 Correlation between spectral features and Shooting Performance Metrics. (A) Correlation between relative band power features and Shooting Performance Metrics. (B) Correlation between ERD/ERS features and Shooting Performance Metrics. All results underwent FDR correction; * denotes p<0.05.

Fig.S 2 illustrates the correlations between spectral features and self-report measures. In the theta band, during the third window, the relative spectral power of the Hostage-Rescue Condition over the frontal region was significantly positively correlated with expectation, and the relative spectral power of the Close-Range Condition over the frontal region was significantly positively correlated with difficulty.

Fig.S 3 presents the correlations between functional connectivity and shooting-performance metrics, as well as between functional connectivity and self-report measures. RTV under the Long-Range Condition was significantly negatively correlated with frontal–parietal functional connectivity in the theta band and significantly negatively correlated with frontal–occipital functional connectivity in the beta band. TIRE under the Hostage-Rescue Condition was significantly negatively correlated with frontal–parietal functional connectivity in the theta band. Difficulty under the Hostage-Rescue Condition was significantly positively correlated with frontal–parietal functional connectivity in the theta band. Fatigue under the Hostage-Rescue Condition was significantly positively correlated with frontal–parietal, frontal–occipital and frontal–frontal functional connectivity in the theta band. Effort under the Close-Range Condition was significantly positively correlated with temporal–occipital functional connectivity in the theta band. Difficulty under the Close-Range Condition was significantly positively correlated with frontal–temporal functional connectivity in the alpha band. Expectation under the Close-Range Condition was significantly positively correlated with frontal–parietal functional connectivity in the alpha band.


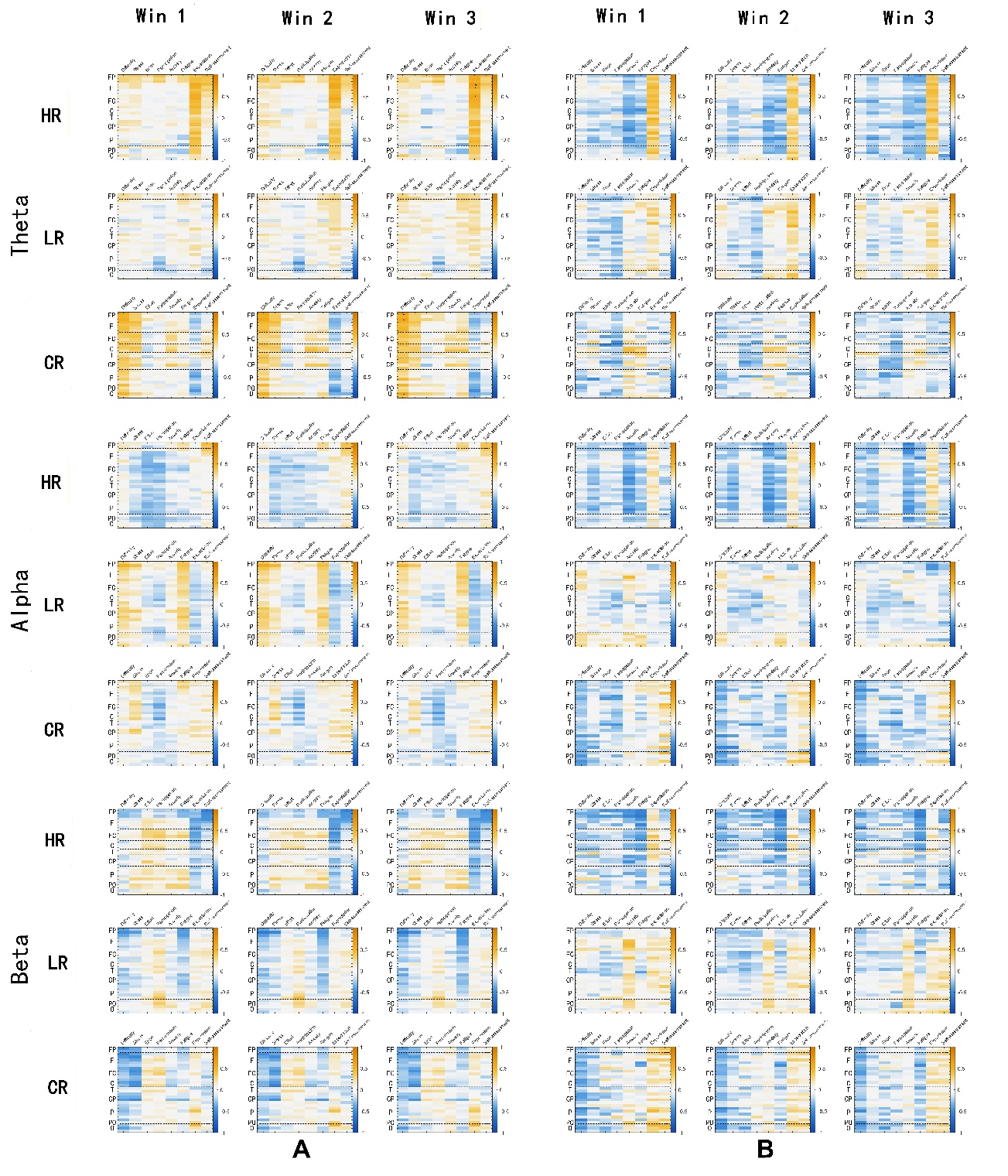


Fig.S 2 Correlation between spectral features and Self-Report Measures. (A) Correlation between relative band power features and Shooting Performance Metrics. (B) Correlation between ERD/ERS features and Shooting Performance Metrics. All results underwent FDR correction; * denotes p<0.05.


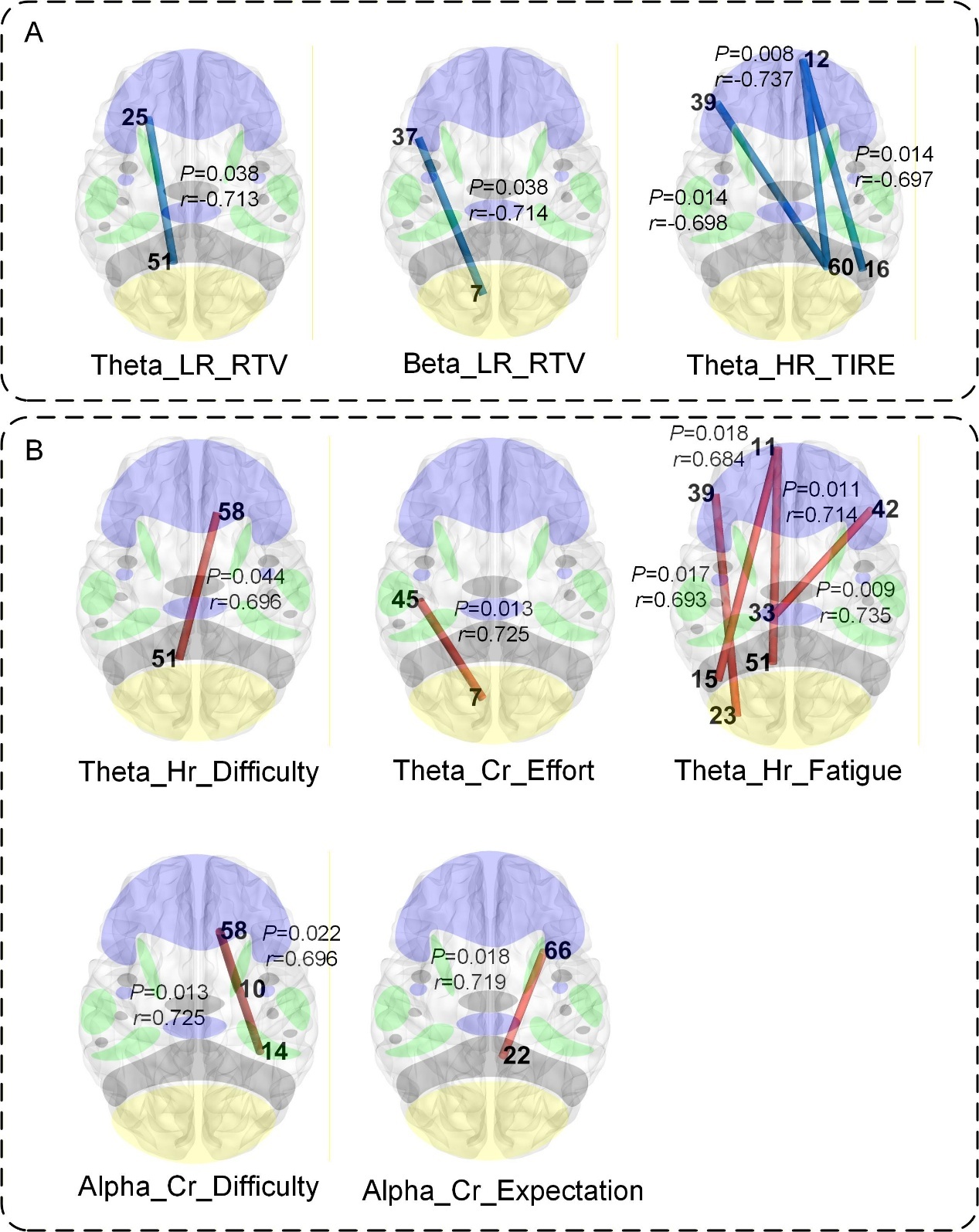


Fig.S 3 Correlation results between functional connectivity and Shooting Performance Metrics and self-report Measures. (A) Correlation results between functional connectivity and shooting Performance Metrics. (B) Correlation results between functional connectivity and self-report Measures. HR denotes Hostage-Rescue Condition, LR denotes Long-Range Condition, CR denotes Close-Range Condition. COG represents center-of-gravity aiming metric, TIRE denotes trigger-pull quality, Anxiety denotes anxiety level, Expectation denotes alignment between actual shooting performance and expectations, Self-assessment denotes self-evaluation of shooting ability. Red indicates significant positive correlations, blue indicates significant negative correlations. Brain region color coding: purple represents frontal lobe, green represents temporal lobe, gray represents parietal lobe, yellow represents occipital lobe. All results underwent FDR correction with a significance level of 0.05.

Table. S 1 presents the correlations between graph-theoretical metrics and self-report measures. Notably, no significant correlations were observed between shooting-performance metrics and any graph-theoretical metrics, whether global or nodal. Among the self-report measures, difficulty exhibited the greatest number of significant associations with global metrics: difficulty was significantly positively correlated with the clustering coefficient and global efficiency of the Hostage-Rescue Condition in both the alpha and theta bands.

Table. S 1 Correlations between graph-theoretical metrics and self-report measures. All results underwent FDR correction with a significance level of 0.05.

| Metrics | Global Metrics | *r* | *p* | Metrics | Nodal Metrics | *r* | *p* |
| --- | --- | --- | --- | --- | --- | --- | --- |
| Difficulty | Theta_Hr_aCp | 0.622 | 0.003 | Difficulty | Alpha_Hr_aBC_RIO_56 | -0.671 | 0.036 |
|  | Theta_Hr_aEg | 0.587 | 0.006 |  | Theta_Hr_aNCC_RIO_68 | 0.793 | 0.000 |
|  | Alpha_Hr_aCp | 0.599 | 0.005 | Stress |  | 0.678 | 0.010 |
|  | Alpha_Hr_aEg | 0.600 | 0.005 | Fatigue |  | 0.717 | 0.003 |
| Fatigue | Alpha_Lr_aCp | 0.542 | 0.019 | Anxiety | Alpha_Cr_aNE_RIO_44 | -0.677 | 0.030 |
