# Supplementary material for: Neural Mechanisms of Shooting Preparation Under High‐Risk and High‐Precision Tasks: A Multiscale EEG Study
Source: Brain Behav. 2026 Mar 9;16(3):e71261. doi: 10.1002/brb3.71261 (PMC12971187; doi:10.1002/brb3.71261)
Supplement: Supplementary file 16 — Supplementary Material: brb371261‐sup‐0016‐SuppMat.docx [file BRB3-16-e71261-s005.docx]

# Shooting Experiment Questionnaire

**Instructions**: This questionnaire assesses your subjective experience during the shooting experiment. The experiment comprises three separate conditions. Complete the corresponding section immediately after finishing each condition, answering on the basis of your genuine feelings.

Questionnaire ID: _______ Participant Name: ________ Age: ________

Date: ___ / ___ / ___

**[Condition One]**

Q1. Difficulty of this shooting session

1 None 2 Very low 3 Low 4 Moderate 5 Moderately high 6 High 7 Very high

Q2. Level of pressure during this shooting session

1 None 2 Very low 3 Low 4 Moderate 5 Moderately high 6 High 7 Very high

Q3. Level of effort during this shooting session

1 None 2 Very low 3 Low 4 Moderate 5 Moderately high 6 High 7 Very high

Q4. Level of engagement during this shooting session

1 None 2 Very low 3 Low 4 Moderate 5 Moderately high 6 High 7 Very high

Q5. Level of anxiety during this shooting session

1 None 2 Very little 3 Slightly 4 Moderate 5 Fairly 6 Quite 7 Extremely

Q6. Level of fatigue after this round of shooting

1 None 2 Very little 3 Slightly 4 Moderate 5 Fairly 6 Quite 7 Extremely

Q7. Did your shooting performance in this round meet your expectations?

1 Far below 2 Below 3 Slightly below 4 Met 5 Slightly above 6 Above  7 Far above

Q8. Please rate your shooting skill level

1 Very poor 2 Poor 3 Below average 4 Average 5 Above average 6 Good 7 Very good

**[Condition Two]**

Q1. Difficulty of this shooting session

1 None 2 Very low 3 Low 4 Moderate 5 Moderately high 6 High 7 Very high

Q2. Level of pressure during this shooting session

1 None 2 Very low 3 Low 4 Moderate 5 Moderately high 6 High 7 Very high

Q3. Level of effort during this shooting session

1 None 2 Very low 3 Low 4 Moderate 5 Moderately high 6 High 7 Very high

Q4. Level of engagement during this shooting session

1 None 2 Very low 3 Low 4 Moderate 5 Moderately high 6 High 7 Very high

Q5. Level of anxiety during this shooting session

1 None 2 Very little 3 Slightly 4 Moderate 5 Fairly 6 Quite 7 Extremely

Q6. Level of fatigue after this round of shooting

1 None 2 Very little 3 Slightly 4 Moderate 5 Fairly 6 Quite 7 Extremely

Q7. Did your shooting performance in this round meet your expectations?

1 Far below 2 Below 3 Slightly below 4 Met 5 Slightly above 6 Above  7 Far above

Q8. Please rate your shooting skill level

1 Very poor 2 Poor 3 Below average 4 Average 5 Above average 6 Good 7 Very good

**[Condition Three]**

Q1. Difficulty of this shooting session

1 None 2 Very low 3 Low 4 Moderate 5 Moderately high 6 High 7 Very high

Q2. Level of pressure during this shooting session

1 None 2 Very low 3 Low 4 Moderate 5 Moderately high 6 High 7 Very high

Q3. Level of effort during this shooting session

1 None 2 Very low 3 Low 4 Moderate 5 Moderately high 6 High 7 Very high

Q4. Level of engagement during this shooting session

1 None 2 Very low 3 Low 4 Moderate 5 Moderately high 6 High 7 Very high

Q5. Level of anxiety during this shooting session

1 None 2 Very little 3 Slightly 4 Moderate 5 Fairly 6 Quite 7 Extremely

Q6. Level of fatigue after this round of shooting

1 None 2 Very little 3 Slightly 4 Moderate 5 Fairly 6 Quite 7 Extremely

Q7. Did your shooting performance in this round meet your expectations?

1 Far below 2 Below 3 Slightly below 4 Met 5 Slightly above 6 Above  7 Far above

Q8. Please rate your shooting skill level

1 Very poor 2 Poor 3 Below average 4 Average 5 Above average 6 Good 7 Very good

—— Thank you for completing the survey! ——
