# Supplementary figures and images for: Neural Mechanisms of Shooting Preparation Under High‐Risk and High‐Precision Tasks: A Multiscale EEG Study
Source: Brain Behav. 2026 Mar 9;16(3):e71261. doi: 10.1002/brb3.71261 (PMC12971187; doi:10.1002/brb3.71261)

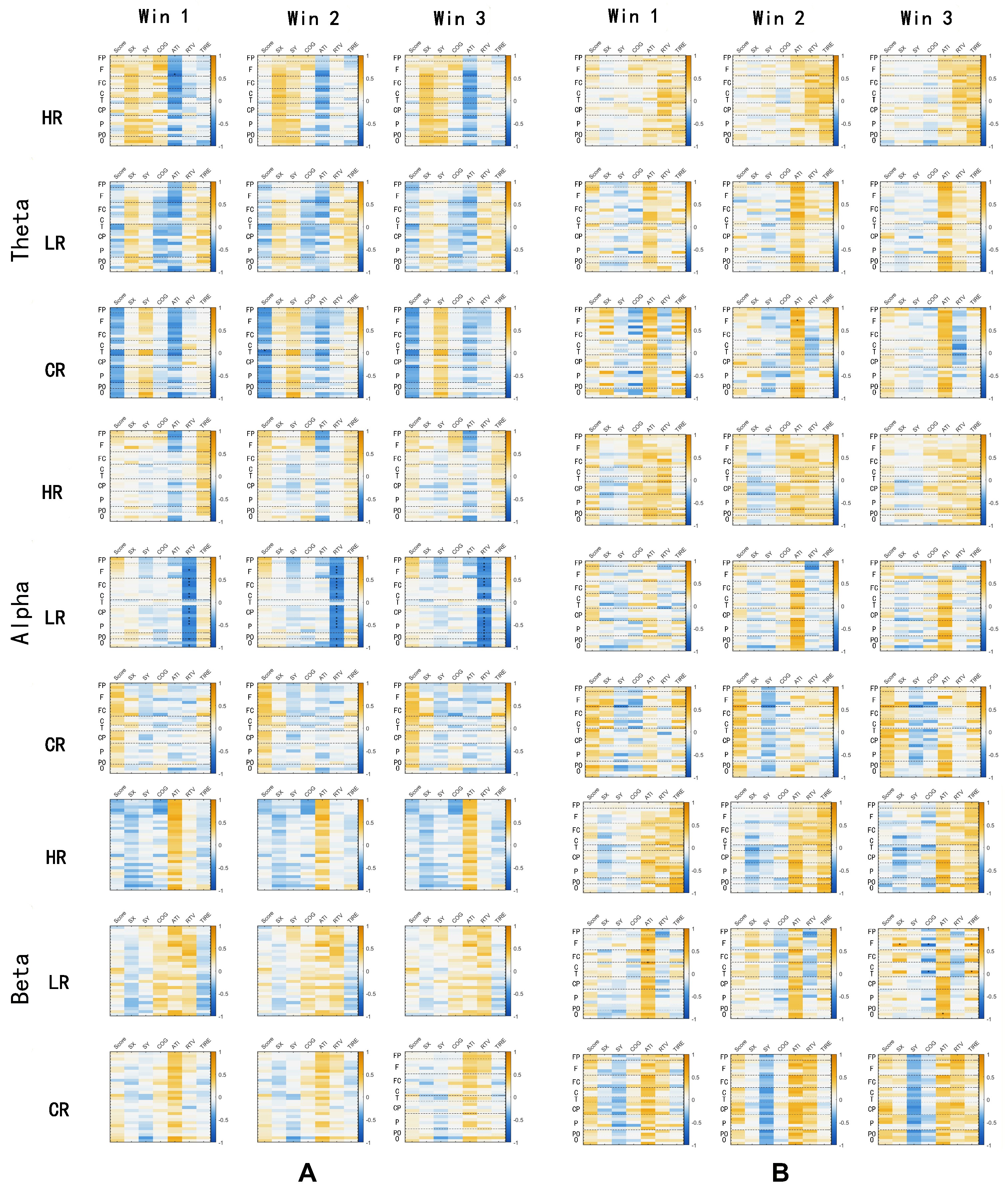

Supplement: Supplementary file 17 — Supplementary Figure: brb371261‐sup‐0017‐FigureS1.jpg [file BRB3-16-e71261-s014.jpg]

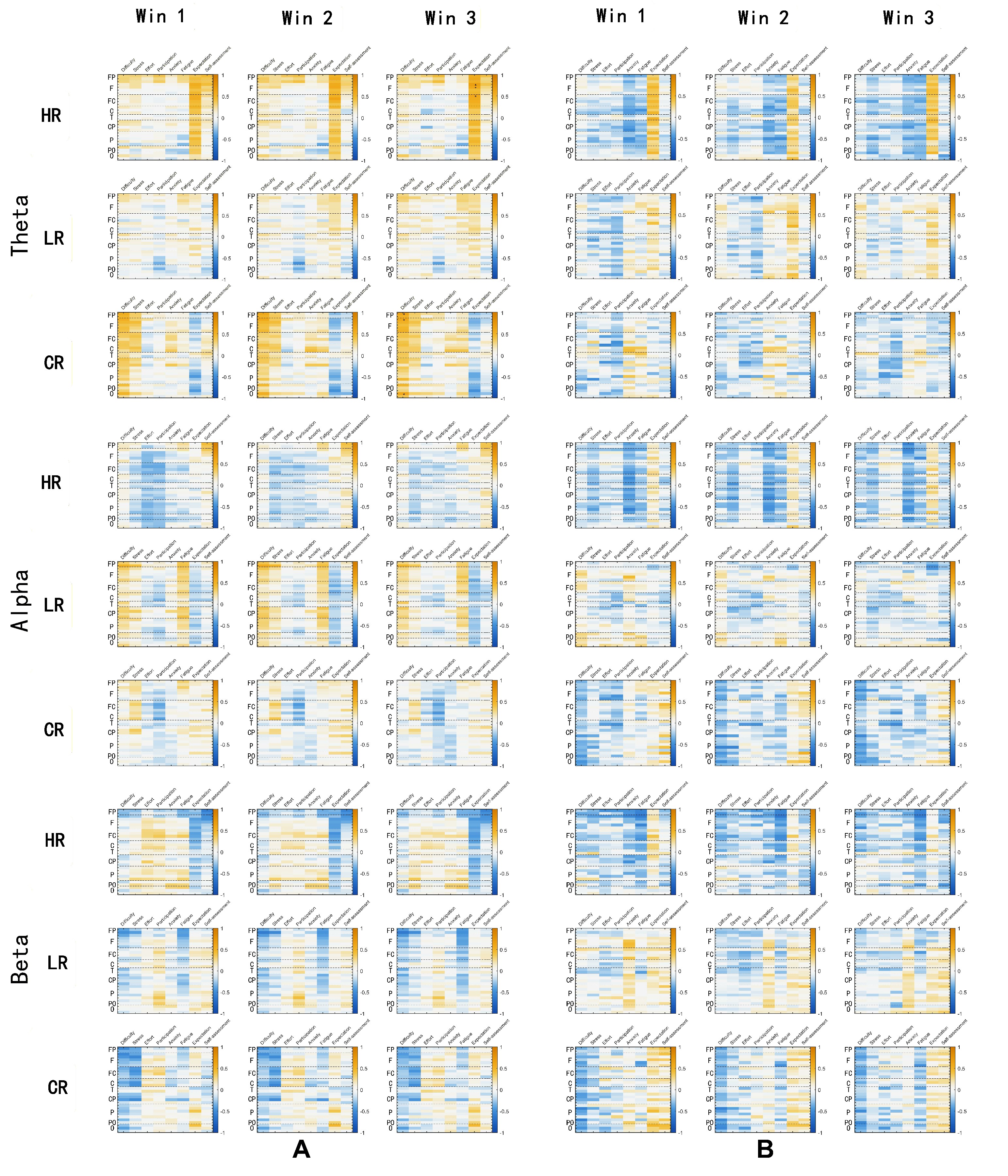

Supplement: Supplementary file 18 — Supplementary Figure: brb371261‐sup‐0018‐FigureS2.jpg [file BRB3-16-e71261-s016.jpg]

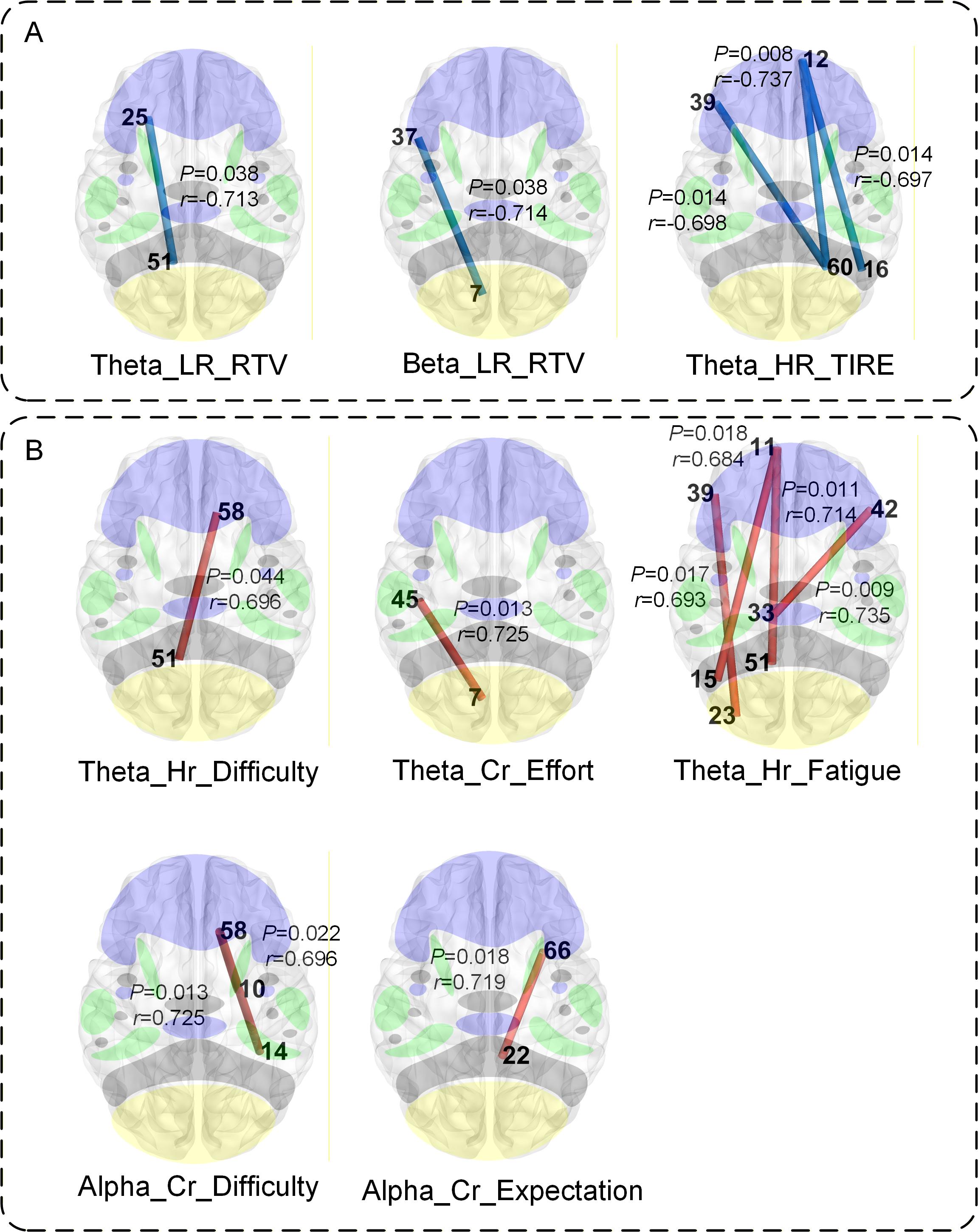

Supplement: Supplementary file 19 — Supplementary Figure: brb371261‐sup‐0019‐FigureS3.jpg [file BRB3-16-e71261-s008.jpg]
